# Fuzzy evaluation and explainable machine learning for diagnosis of rheumatic and autoimmune diseases

**DOI:** 10.7717/peerj-cs.3096

**Published:** 2025-08-11

**Authors:** Mohammed Fadhil Mahdi, Arezoo Jahani, Dhafar Hamed Abd

**Affiliations:** 1 Faculty of Electrical and Computer Engineering, Sahand University of Technology, Tabriz, Iran; 2 College of Computer Science and Information Technology, University of Anbar, Anbar, Iraq

**Keywords:** Explainable, Decision making, Fuzzy decision by opinion score method (FDOSM), Fuzzy evaluation, Rheumatic and autoimmune diagnosis, Machine learning

## Abstract

In this article, a new combination of an explainable machine learning approach with a fuzzy evaluation framework is proposed to improve the diagnostic performance and interpretation of rheumatic and autoimmune diseases. This work addresses three major challenges: (i) overlapping symptoms and complex clinical presentations, (ii) the lack of interpretability in traditional machine learning models, and (iii) the difficulty of selecting the best diagnosis model. To overcome these challenges, a new dataset was collected from Iraq’s hospitals and health centers between 2019 and 2024. The size of dataset is 12,085 patients and includes 14 features in seven classes (rheumatoid arthritis, reactive arthritis, ankylosing spondylitis, Sjogren syndrome, systemic lupus erythematosus, psoriatic arthritis, and normal). The dataset is subjected to extensive preprocessing with attribute imputation (mean and mode), encoding categorical features, and balancing the data to pass it to 12 different machine learning models. Performance is evaluated based on precision, recall, F-score, kappa, Hamming loss, Matthews correlation coefficient, and accuracy to identify the best model. To select the optimal model, we apply fuzzy decision by opinion score method (FDOSM). The FDOSM process involves assessments from three domain experts to ensure a robust and well-rounded evaluation. Furthermore, the explainable artificial intelligence (XAI) technique provides global and local explanations for model predictions. Local interpretable model explanations (LIME) were used as explanations and significantly increased the transparency and reliability of the clinical decision-making process. The results show that the FDOSM yields gradient boosting with a 0.1333 score and a rank of 1, is the best model with an accuracy of 86.89%, precision of 87.35%, and kappa of 84.51%. The best model using XAI to increase confidence and trustworthiness in clinical decision-making and healthcare applications.

## Introduction

Rheumatic and autoimmune diseases pose significant diagnostic challenges due to overlap of symptoms, variable progression of the disease, and the absence of specific biomarkers. These complexities often result in delayed or inaccurate diagnoses, affecting patient outcomes. To address the lack of this, we propose a new explainable machine learning framework that integrates FDOSM with XAI techniques for enhanced diagnostic accuracy and transparency. The framework is applied to a newly developed multiclass clinical dataset collected from Iraqi hospitals and health centers between 2019 and 2024, covering six common rheumatic and autoimmune diseases with normal (Mahdi, Jahani & Abd, 2025). This work aims to provide clinicians with an intelligent, explainable tool that supports more informed diagnostic decisions in healthcare. Rheumatic and autoimmune diseases are chronic diseases that can cause irreversible pathology in various organs, especially when diagnosis late (Ballestar, Sawalha & Lu, 2020). Early and accurate diagnosis of these diseases is crucial for proper treatment and for controlling the morbidity and mortality associated with these types of diseases (Wang et al., 2021). Studies indicate that the incidence of rheumatic increased by approximately 14.1% between 1990 and 2020 (Li, Yuan & Ou, 2024). In 2020, an estimated 17.6 million people worldwide lived with RA, and this number is projected to increase to 31.7 million by 2050 (Giacomelli et al., 2021). Autoimmune diseases affect approximately 5–10% of the population in industrialized countries, and this rate is increasing (Miller, 2023). Currently, the diagnosis of these diseases is made by experienced professionals based on the clinical findings of the patients and specific blood tests, in which the presence of some antibodies and clinical manifestations are positive and negative, respectively (Lenti et al., 2022).

Rheumatic and autoimmune diseases represent a substantial number of chronic and enabling diseases associated with inflammation and immune system dysregulation. It is divided into noninflammatory and inflammatory joints (Sharma & Sharma, 2022). Noninflammatory diseases include ordinary degenerative diseases, such as osteoarthrosis, osteoarthropathy, and senile osteoporosis, among others. Inflammatory pain can be divided into infectious and noninfectious types (Bentaleb et al., 2020). Noninfectious inflammatory diseases include rheumatic and autoimmune diseases, such as rheumatoid arthritis, ankylosing spondylitis, and reactive arthritis, among others, whereas infectious rheumatic diseases are acute rheumatic fever (Scherer, Häupl & Burmester, 2020). Conditions such as rheumatoid arthritis, Sjögren’s syndrome, and systemic lupus erythematosus pose major challenges for diagnosis because of similarities in symptoms, different disease courses, and contamination of several cases due to the absence of particular biomarkers (Zeidler & Hudson, 2021; Radu & Bungau, 2021). Rheumatic and autoimmune diseases often present multiple challenges due to their complexity, which is further compounded by patients who may present in various ways (Madrid-García et al., 2023). More advanced diagnostic instruments are needed to make more accurate diagnoses.

Early diagnosis is necessary to control the progression of the disease, perform treatment to avoid irreversible damage, and achieve better outcomes for patients (Almutairi et al., 2021). However, such traditional approaches to diagnostic procedures are often based on the experience and interpretation of the test results, which may not always produce the correct results or could contribute to substantial delays in starting treatment in some patients. Today, this problem is solved by the availability of a large amount of medical data. Artificial intelligence (AI) has become an increasingly valuable tool in medical diagnosis. In particular, machine learning (ML) has achieved significant success in classification and prediction problems and has also been adopted in the detection and diagnosis of rheumatic and autoimmune diseases. This work focuses on using ML for tabular data due to the advantages (Ye et al., 2024; Shwartz-Ziv & Armon, 2022; Borisov et al., 2022), such as (i) lower computational power, (ii) explainability, (iii) working well with small datasets, (iv) fewer hyperparameters, (v) handling heterogeneous data, and (vi), fast training.

The multi-criteria decision-making (MCDM) for the ML model for diagnosing rheumatic and autoimmune diseases can be distinguished by utilizing more than the accuracy of the learning model for the optimization requirement (Kumar et al., 2022; Taherdoost & Madanchian, 2023). The importance of the typical goal is to find the best classification model to classify diseases with high accuracy. This work focuses on the best trade-off model by combining and performing model selection after the classification task. Hence, the MCDM problem is explained by evaluating the 12 different learning models are evaluated according to seven different evaluation criteria for selecting the best multi-criteria model. Despite the effectiveness of the MCDM method, it has several challenges and drawbacks (Liu, Eckert & Earl, 2020). However, in order to resolve these issues, one would require a more inclusive solution. More recently, Liu, Eckert & Earl (2020) introduced quite a revolutionary approach to MCDM methods, which they termed as fuzzy decision by opinion score method (FDOSM) which is significantly more effective than existing MCDM methods. The FDOSM mechanism relies on the formulation of opinion matrices based on expert assessments. The experts identify the optimum value and use it as a benchmark against other parameters for the relevant criteria across the alternatives. Thereafter, once the opinion matrix has been established, the final rank for alternatives is established through simple direct aggregation using the arithmetic mean. Two different contexts form the basis of the FDOSM which are individual decision context and group decision context. The FDOSM also incorporates ideal solution concepts, reduces the number of necessary comparisons, eliminates inconsistencies, minimizes vagueness, reduces calculations, and ensures understandable comparisons that are inherently fair. Since the solution relies on expert opinions, FDOSM also results in rational outcomes.

ML has effectively automated diagnosis across various fields, including autoimmune diseases. Utilizing work with a large dataset, ML models can discover intricate patterns and connections hidden within the data, which may be difficult for physicians. However, the major problems faced the researchers in practice of using these models are interpretability and accountability. It is particularly important when diagnosing chronic autoimmune diseases. Understanding how the model makes a decision is crucial because the stakes are very high. This is where explainable AI (XAI) is introduced (Alivernini et al., 2024). XAI improves the interpretability of the decisions made by machine thinking decisions about model understanding and how workers can trust its prediction (Patibandla et al., 2024). In clinical settings, XAI plays a crucial role in enhancing the transparency of diagnostic, building trust among healthcare professionals, and supporting informed decision-making that directly impacts patient outcomes. Furthermore, the use of fuzzy evaluation in ML algorithms addresses uncertain information, which is prevalent in medical data, leading to better clinical decision-making amidst the complicated nature of healthcare.

Many studies have been conducted on rheumatoid disease; some of them have been performed with images and others with text (McMaster et al., 2022; Trottet et al., 2024; Danieli et al., 2023). As summarized in Table 1, existing literature reveals a focus on binary classification, disease-specific models, or limited data types, often lacking interpretability or real-world validation. Our study addresses these gaps by developing an explainable ML model integrated with FDOSM, capable of diagnosing six autoimmune and rheumatic diseases with normal using real clinical data and supporting clinical decision-making with transparency and generalizability. To the best of our knowledge, this is the first work on tabulated data, specifically six of the most common types of rheumatic and autoimmune diseases with normal condition. The main challenge with this work is that there is no dataset covering various rheumatic and autoimmune diseases, including rheumatoid arthritis (RA), reactive arthritis (ReA), ankylosing spondylitis (AS), Sjögren’s syndrome (SS), systemic lupus erythematosus (SLE), and psoriatic arthritis (PA).

**Table 1:** Summary of recent studies on autoimmune disease diagnosis using ML.

Ref	Year	Disease	Method	Dataset (Type, Size)	Explainability	Multi-Class	FDOSM
Martins et al. (2023)	2023	ALS, MS, SLE, CD, AIH	LR, NB, SVM	Synthetic Tabular, 1,000	✗	✓	✗
Martorell-Marugán et al. (2023)	2023	SLE, SS	ML on omics	Tabular, 651	✓	✗	✗
Wang et al. (2023)	2023	LN (subset of SLE)	XGB, LGB, ANN	Tabular, 1,467	✗	✗	✗
Zhang et al. (2023)	2023	RA	Bibliometric	N/A, 859 articles, N/A	✗	✗	✗
Park (2024)	2024	SLE	DT, RF, GB	Tabular, 24,990	✗	✗	✗
Pham et al. (2024)	2024	RA	DT, RF, XGB	Tabular, 5,600	✗	✗	✗
Jia et al. (2024)	2024	AS	bSCJAYA-FKNN	Tabular (UCI), 11 datasets	✗	✗	✗
Nouira et al. (2024)	2024	SS	CNN	Image, 225	✗	✗	✗
Qiu et al. (2025)	2025	RA	LightGBM, RF	Tabular, 1,000	✗	✗	✗
Wu et al. (2025)	2025	RA	10 ML	Tabular, 2,106	✗	✗	✗
Our study	2025	6 diseases with normal	12 ML models + FDOSM + LIME	Tabular, 12,085	✓	✓	✓

This work addresses the significant challenges of creating a reliable, explainable and precise diagnostic model for rheumatology and autoimmune diseases *via* tabular clinical data. Most existing work have focused on limited disease categories, image datasets, and their overlapping clinical presentations. Although traditional ML models stand out in terms of prediction accuracy, they do not explain the rationale for their inclusion, which is a drawback in clinical practice. The primary objective of this work is to develop a comprehensive diagnostic framework that combines ML models with FDOSM and XAI techniques to enhance the accuracy, transparency, and trustworthiness of diagnostic decisions in rheumatology. This study introduces a novel dataset, which was meticulously collected from hospitals and health centers in Iraq over 5 years between 2019 and 2024, covering six major classes of rheumatic and autoimmune diseases with normal condition. The novelty of this research lies in its integration of ML models with FDOSM on tabular clinical data with the use of XAI to provide both global and local interpretability for clinical decision support. To our knowledge, this is the first work that systematically combines these techniques for rheumatic and autoimmune diseases. The following three contributions are made in this work.

– We introduce a novel dataset collected from 2019 to 2024, covering seven classes of rheumatic and autoimmune diseases, including RA, ReA, AS, SS, SLE, PA, and a normal control group.
– Twelve different ML models are tested and evaluated *via* different evaluation metrics, such as accuracy, precision, recall, and kappa score. We apply FDOSM to select the most suitable ML model on the basis of performance and interpretability.
– We implement XAI techniques to provide local and global explanations of the model’s predictions, ensuring that clinicians can trust the diagnostic recommendations.

The remainder of this work is structured as follows: “Proposed Framework” describes the proposed framework, dataset collection, and the preprocessing techniques used, including the imputation of missing data and the hot encoding of categorical variables. Additionally, presents the ML models evaluated in this work, along with the FDOSM methodology for model selection. “Results and Discussion” presents the results, discusses the implementation of XAI techniques and their integration into the diagnostic system. It is also presents the experimental results, comparing the performance of different models and the explainability of the best model. “Policy Suggestions” policy suggestions; finally “Conclusion” concludes the work and discusses the findings, their implications, and potential directions for future work.

## Proposed framework

**Figure 1:**
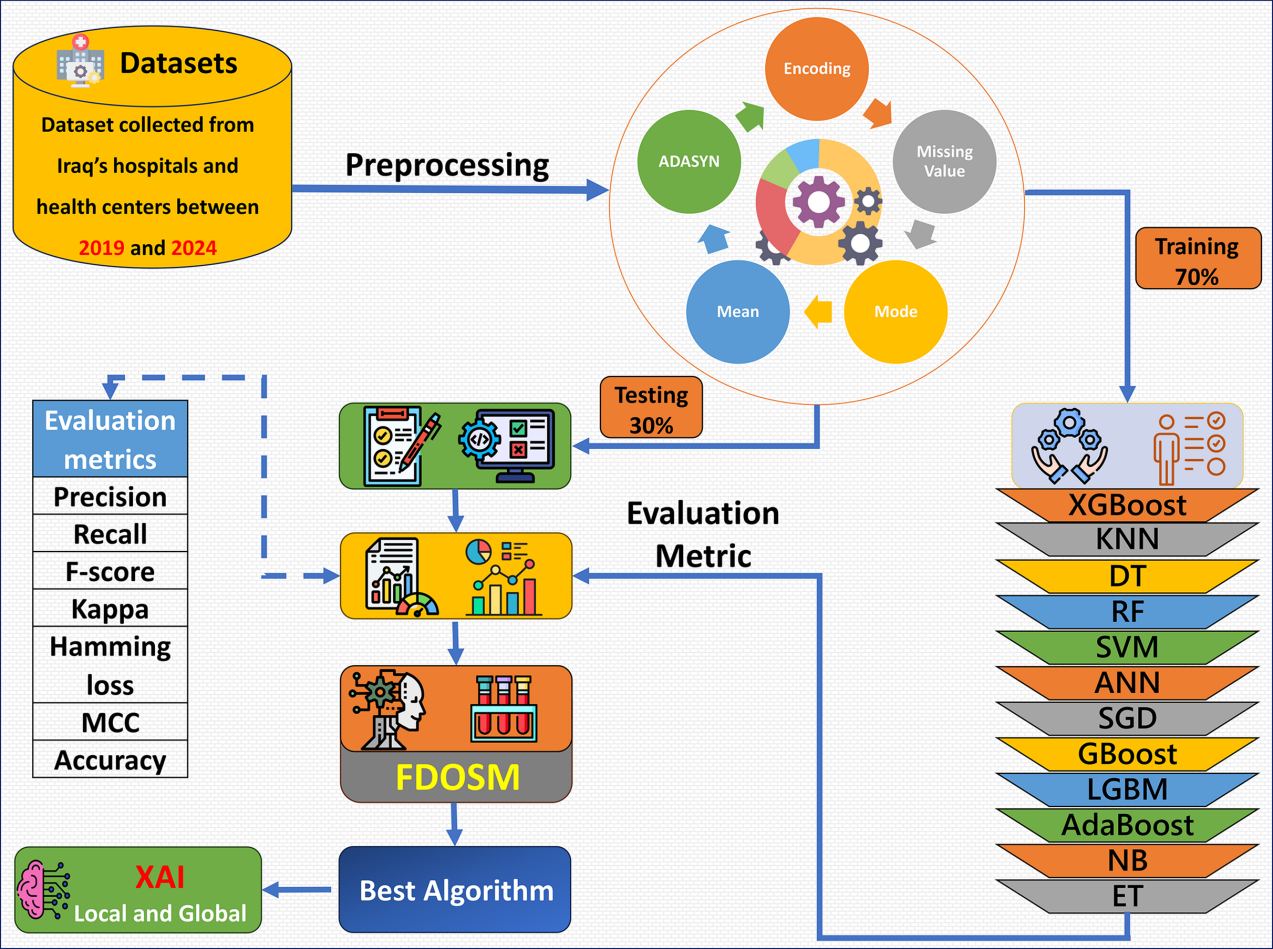
Overview of the proposed framework.

The proposed framework consists of five phases: dataset collection, preprocessing, learning model construction with evaluation metric, FDOSM, and XAI, as shown in Fig. 1. Data were collected from hospital records, electronic health records, and laboratory tests. After the dataset is collected, the preprocessing phase addresses the mean of the numerical feature and mode for categorical feature imputation for missing values, encoding of the categorical features, and up sampling the minority class *via* Adaptive Synthetic Sampling Approach (ADASYN). Then 12 ML algorithms are trained, such as XGBoost, random forest (RF), gradient boost (GBoost), light gradient boost machine (LGBM), and support vector machine (SVM). The fitted models are tested, and an assessment is performed with the use of precision, recall, F-score, kappa, hamming loss, MCC, and accuracy. After these phases, the FDOSM model rank the obtained values on the basis of the calculated metrics, highlighting the best performing algorithms concerning the task in question. This ensures that there is a stepwise procedure for how the model is trained, tested, and restricted for predictive tasks within the health domain.

This research offers significant societal benefits by addressing the challenges in early and accurate diagnosis of rheumatic and autoimmune diseases, which are often underdiagnosed or misdiagnosed due to their overlapping symptoms and complex nature. By integrating ML and FDOSM XAI, this work provides a robust, interpretable, and data-driven decision support for clinicians. Key benefits include improved diagnostic accuracy, enhanced interpretability of AI-driven decisions, strengthened healthcare infrastructure through reliable decision aids, and ultimately, better patient outcomes through timely and precise diagnoses.

### Dataset collection

The problem of diagnosing rheumatic and autoimmune diseases to avoid sample bias is essential for obtaining an extensive and well-organized dataset to ensure that the model is optimal for all types of patients and diseases. Our work data were collected between 2019 and 2024 from various medical factors in Iraq, including one hospital and three laboratories of a wide range of autoimmune conditions, confirming variety in the subjects of the work in terms of familial and disease presentation (Mahdi, Jahani & Abd, 2025; Abd, Mahdi & Jahani, 2025). Special care was taken to ensure that the dataset was diverse and anonymous to facilitate the privacy of the patients while allowing for successful training and model evaluation. This study was approved by the Saint Raphael Hospital and Al-Hawraa, Al-Sibtain, and Taqadum health centers under approval numbers 25, 66, 98, and 122, respectively. Table 2 lists 14 features corresponding to the rheumatic and autoimmune diseases.

**Table 2:** Description of numerical and categorical features used in autoimmune disease prediction.

No	Feature type	Feature name	Description
1	Numerical	Age	Represents the age of the patients in years.
2		ESR	Erythrocyte sedimentation rate (ESR) measures how quickly red blood cells settle at the bottom of a test tube. Higher levels indicate inflammation
3		CRP	C-reactive protein (CRP) is a blood marker of inflammation. High CRP levels can indicate inflammation or infection.
4		RF	Rheumatoid factor (RF) is an antibody found in the blood, commonly elevated in individuals with rheumatoid arthritis and autoimmune diseases.
5		Anti-CCP	Anti-cyclic citrullinated peptide (Anti-CCP) is an antibody found in the blood, often present in patients with rheumatoid arthritis, helping in early diagnosis.
6		Anti-dsDNA	Anti-double stranded DNA (Anti-dsDNA) antibodies are highly specific to SLE and used for diagnosis and monitoring.
7		Anti-Sm	Anti-Smith (Anti-Sm) antibodies are specific for SLE, and their presence helps confirm diagnosis.
8		C3	Complement component 3 (C3) is part of the immune system. Low levels may indicate autoimmune diseases or infections.
9		C4	Complement component 4 (C4) works in conjunction with C3 in immune responses. Reduced levels are often seen in autoimmune disorders.
10	Categorical	Gender	The biological sex of the individual (Male/Female). Gender is often considered when analyzing disease prevalence or response to treatment.
11		HLA-B27	Human leukocyte antigen B27 (HLA-B27) is a genetic marker associated with autoimmune diseases like ankylosing spondylitis.
12		ANA	Antinuclear antibodies (ANA) are a group of antibodies that target substances in the nucleus of cells. High ANA levels can be a sign of autoimmune disorders like lupus.
13		Anti-Ro	Anti-Ro antibodies are primarily associated with autoimmune conditions like Sjögren’s syndrome and lupus.
14		Anti-La	Anti-La antibodies are often found in patients with Sjögren’s syndrome and lupus, indicating an autoimmune reaction.

Table 3 shows the patient distributions of certain rheumatic and autoimmune diseases and the normal healthy population. Among these, RA is the most abundant with 2,848 cases, making this work even better with minimum cases, as it is proven to be easier to obtain in an information system. AS and SS also have a reasonable number of cases (2,127 and 1,852, respectively), which helps to model the relevant conditions. In total, 1,783 and 1,604 cases of PA and N, respectively, have been reported, which still provides sufficient cases for classification even with the intricacy of the disease. The SLE class (1,355 cases) is necessary for helper states since it helps to distinguish between healthy and diseased states, reducing the chances of false positives. Although the smallest ReA class with only 516 cases is faced with many challenges regarding the model’s ability to identify its characteristics. This class is imbalance, particularly the small size of the ReA class compared with the RA class, might have a negative effect on the performance of the model in terms of discrimination, especially where these diseases are more common and thus expected. To overcome this problem, methods such as resampling, class sparsity, and data enhancement for class imbalance is applied so that the model performs equally well in all diseases, even those that are rare, such as ReA.

**Table 3:** Number of samples for each disease category.

Disease	Number size
RA	2,848
AS	2,127
SS	1,852
PA	1,783
N	1,604
SLE	1,355
ReA	516

### Preprocessing

One of the most important stages in the process of analyzing any data is the preprocessing, as it enables the model to perform well (Zhu, Jiang & Alonso, 2024). It encompasses several steps that aim to cleansing, converting, and structuring the data in a manner that increases the efficiency of the ML algorithms. Three preprocessing steps were used in this work:

– Missing values are handled *via* the mean and mode (Palanivinayagam & Damaševičius, 2023). The mean imputation for numerical variables is shown in Eq. (1). This method is useful only if the data are missing in some cases owing to randomness and if the population is approximately normal. Additionally, for categorical variables, the missing values are imputed with the mode, that is, the maximum occurring category, as shown in Eq. (2)
(1) $${m_{i}} = {{\sum\nolimits_{j = 1}^n {{m_{j}}} } \over n}\quad{{\mathrm{for}}}\;{m_{j}} \in \{ {m_{1}},{m_{2}},{m_{3}}, \ldots ,{m_{j}}\} $$
(2) $${\beta _{mode}} = \mathop {{{\mathrm{argmax}}}\;{{\mathrm{frequency}}}({\beta _{{\mathrm{k}}}})}\limits_{{\beta _{k}}}$$
where $m_i$ is the missing value, $\sum\nolimits_{j = 1}^n {{m_{j}}}$ is the sum of all nonmissing values, $n$ is the number of nonmissing values, ${\beta _{mode}}$ is the most frequent category, and ${\beta _{k}}$ represents each distance category in the category feature.
– ML approaches tend to be more efficient when applied to numeric inputs; hence, the need to encode categorical variables into numbers, for example, is recommended. We use one-hot encoding. In which each category value is represented by a binary vector, where only one element is 1 and the other is 0, as shown in Eq. (3).
(3) $$One{\hbox{-}}Hot({C_{i}}) = [{b_{1}},{b_{2}},{b_{3}}, \ldots ,{b_{n}}]\;where\;{b_{j}} = \left\{ {\matrix{ {1\quad if\quad j = i} \cr {0\quad otherwise} \cr } } \right.$$
where *C* is the categorical variable, $n$ is the total number of unique *C*, and $b$ is the binary vector.
– In medical datasets for definition and classification, targets with imbalanced classes (*e.g*., rare diseases) are usually available, and for such cases, the adaptive synthetic sampling (ADASYN) is said to be an effective method for creating synthetic samples of the minority class (Khan et al., 2021; Davagdorj et al., 2020). ADASYN focuses on instances that are difficult to learn and modifies itself according to the distribution of the data. This method address the class imbalance problem without generating uniform oversampling, resulting in optimal representations of the minor classes. This method prevents bias toward common classes and improves rare disease analysis. The following steps for ADASYN algorithm:
Let the training dataset be $D = \{ ({x_{i}},{y_{i}})\} _{i = 1}^n$, where ${x_{i}} \in {\mathbb R}^{d}$ and ${y_{i}} \in \{ 0,1\}$, with class one as the minority class.
  1. **Identify minority class:**
  Let $m$ be the number of minority class examples. Define $\beta$ as the desired balance level, and compute the total number of synthetic samples to be generated, as shown in Eq. (4).
(4) $$G = ({n_{\rm {maj} }} - {n_{\rm {min} }}) \times \beta$$
  where ${n_{{{\mathrm{maj}}}}}$ and ${n_{{{\mathrm{min}}}}}$ are the number of majority and minority samples, respectively.
  2. **Compute local imbalance per minority sample:**
  For each minority sample ${x_{i}}$, find its $k$-nearest neighbors ( $k = 5$) from the entire dataset. Let ${\eta _{i}}$ be the ratio of majority samples among the $k$ neighbors of ${x_{i}}$, as shown in Eq. (5).
(5) $${\eta _{i}} = {{ {\rm total\;of\;majority\;samples\;among\;} k{\mathrm{\;of\;}} {x_{i}}} \over k}.$$
  Normalize the ratios to obtain ${r_{i}}$, by using Eq. (6).
(6) $${r_{i}} = {{{\eta _{i}}} \over {\sum\nolimits_{j = 1}^m {{\eta _{j}}} }}.$$
  3. **Determine number of synthetic samples per instance:**
  Calculate the number of synthetic samples to be generated for each ${x_{i}}$, using Eq. (7).
(7) $${g_{i}} = {r_{i}} \cdot G .$$
  This ensures that more synthetic data is generated for those minority samples surrounded by more majority samples.
  4. **Generate synthetic samples:**
  For each ${x_{i}}$, randomly select one of its $k$-nearest minority neighbors ${x_{zi}}$, and generate synthetic samples using the following interpolation, as shown in Eq. (8).
(8) $${x_{\rm {new} }} = {x_{i}} + \delta \cdot ({x_{zi}} - {x_{i}}),\quad \delta \sim {\cal U}(0,1)$$
  Repeat this for ${g_{i}}$ times to create the required number of synthetic samples for ${x_{i}}$. In this work ReA class increased from 516 to 2,946 samples.

### Model training

In this section, after preprocessing, the data is moved to the data training, and different algorithms are practiced. The selection of the 12 ML models was based on their established performance in medical classification tasks that involved tabular data. These models cover a broad spectrum of algorithmic types, including ensemble learning (RF, XGBoost, LGBM, GBoost, AdaBoost, extra trees (ET)), probabilistic learning naive Bayes (NB), distance-based methods k-nearest neighbors (KNN), linear classifiers (SVM, SGD), decision tree (DT), and artificial neural networks (ANN). This diversity allows for comprehensive benchmarking across different algorithmic families. Each of these algorithms is trained on the dataset, and then the different performances are evaluated.

Let us assume that we have dataset *D* and this dataset will split into training and testing sets. Both sets have the characteristics $X = x1,x2,x3, \ldots ,xi$, and we have the labels $Y = y1,y2,y3, \ldots ,yj$. The formula becomes as follows:

(9) $$D = {x_{i}},{y_{j}}|i = 1,2,3, \ldots ,n,j = 1,2,3, \ldots ,m .$$

In this work, *D* is divided into two sets, ${D_{train}}$ and ${D_{test}}$, using ratios of 70% ${D_{train}}$ and 30% ${D_{test}}$ for each class, as shown in Table 4.

**Table 4:** Training and test set sizes for each disease category.

Disease	${ D_{train}}$ size	${ D_{test}}$ size	Total
RA	1,997	851	2,848
AS	1,474	653	2,127
SS	1,318	534	1,852
PA	1,242	541	1,783
N	1,125	479	1,604
SLE	918	437	1,355
ReA	2,086	860	2,946
**Total**	10,160	4,355	14,515

For training set ${D_{train}}$, the aim is to optimize the model parameters to minimize the error between the prediction $\widehat y$ and the actual value $y$. Equation (10) shows how our models are trained.

(10) $${\theta ^*} = \mathop {\arg min}\limits_\theta \;\;\psi ({g_\theta }(x),y)$$

where $\theta$ represents the model parameters, ${g_\theta }$ represents the model prediction with the parameters, $\psi$ is the loss function for the model, $y$ is the actual label, and ${\theta ^*}$ represents the parameter optimization that was obtained during training, as shown in Table 5.

**Table 5:** Parameter settings for all ML algorithms.

Models	Parameter values
XGBoost	estimators = 100, depth = 6, learning_rate = 0.3, $\gamma = 0,\alpha = 0,\lambda = 1$
KNN	neighbors = 5, weights = ‘uniform’, distance = Minkowski
DT	Criterion = ‘gini’, splitter = ‘best’, split = 2
RF	estimators = 100, criterion = ‘gini’, split = 2
SVM	C = 1.0, kernel = ‘rbf’, tol = 0.001
ANN	hidden = 100, activation = ‘relu’, solver = ‘adam’, learning_rate = 0.2, $\alpha = 0.0001$, iter = 200
SGD	Penalty = l2, $\alpha = 0.0001$, learning_rate = 0.2, iter = 1000, tol = 0.001
GBoost	estimators = 100, learning_rate = 0.1, depth = 3, split = 2
LGBM	estimators = 100, learning_rate = 0.1, leaves = 31, child = 20
AdaBoost	estimators = 50, learning_rate = 1.0
NB	var_smoothing = 1e−9
ET	estimators = 100, criterion = ‘gini’, split = 2, leaf = 1

To mitigate overfitting in GBoost and XGBoost we increased regularization $\lambda$ = 0.2. For ANN algorithm Added dropout (rate = 0.2) and implemented early stopping (patience = 100 iter). Finaly, reduced max tree depth from 6 to 4 and feature subsampling (50% per tree).

After parameter setting and training our model ${D_{train}}$, the model is tested through ${D_{test}}$. Equation (11) is used for model prediction for the test data.

(11) $${\widehat y_{l}} = {g_\theta }({x_{i}}).$$

To evaluate our model performance base on the prediction $\widehat y$ different metrics were used, such as accuracy, precision, recall, F-score, kappa, MCC, and HL. All these metrics are explained in the next section. Algorithm 1 shows the steps of the learning model.

**Algorithm 1:** Learning model for training and testing sets with prediction.

** Input:** Test and train set size
1 ${D_{train}} = \{ ({x_{i}},{y_{j}})\;\vert\;i = \{ 1,2,3, \ldots ,n\} ,j = \{ 1,2,3, \ldots ,m\} \}$, training set
2 ${D_{test}} = \{ {x_{i}}\vert i = \{ 1,2,3, \ldots ,e\} \}$, test set
3 **Begin**
4 /*Initialization:*/
5 $Y \leftarrow \emptyset$;
6 Read the training *D*_*train*_
7 Setting parameters according to Table 5
8 /*Computation:*/
9 **for** ${d_{i\epsilon }}$ *D*_*train*_ **do**
10 ${\theta ^*} \leftarrow$ learning model using Eq. (10);
11 **for** ${d_{i\epsilon }}{D_{test}}$ **do**
12 **end**
13 (a) ${\widehat y_{l}} \leftarrow$ calculate labels for each class using Eq. (11);
14 (b) $y \leftarrow$ the True label in *D*_*test*_;
15 (c) $Y \leftarrow Y \cup \{ y\} ;$
16 **end**
**Output** Predicted label set
17 Y = $\{{y_{j}}\;|\;j\epsilon \{1,2,3,\ldots,e\}\}$-the test size in *D*_*train*_ that have the predetermined class labels

### Evaluation metrics

In ML, it is essential to examine models to evaluate how well they might perform on a task or whether they are fit for purpose. Different metrics are used to evaluate models on the basis of their prediction accuracy, error types, and ability to extend the model beyond the data used for training. These metrics help to report how well a model performs on some prediction tasks. Table 6 shows the most common evaluation metrics as they relate to model performance (Abd et al., 2023).

**Table 6:** Performance evaluation metrics of ML models.

Metric	Equation
Precision	$\displaystyle {{TP} \over {TP + FP}}$
Recall	$\displaystyle {{TP} \over {(TP + FN)}}$
F-score	$\displaystyle {{2*Precision*Recall} \over {Precision + Recall}}$
Kappa	$\displaystyle {{{p_{o}} - {p_{e}}} \over {1 - {p_{o}}}} = {{(TP + FP)*(TP + FN) + (TN + FN)*(TN + FP)} \over {{N^{2}}}}$
Hamming loss (HL)	$\displaystyle {1 \over N}\sum\nolimits_{i = 1}^N {{{numberofincorrectclassesfor\;i} \over {otalnumberofclassesfor\;i}}}$
Matthews correlation coefficient (MCC)	$(TP*TN) - (FP*FN)$
Accuracy	$\displaystyle {{TN + TP} \over N}$

**Note:**

Here, TP, true positive; TN, true negative; FN, false negative; FP, false positive; and is the total number of samples.

### FDOSM

**Figure 2:**
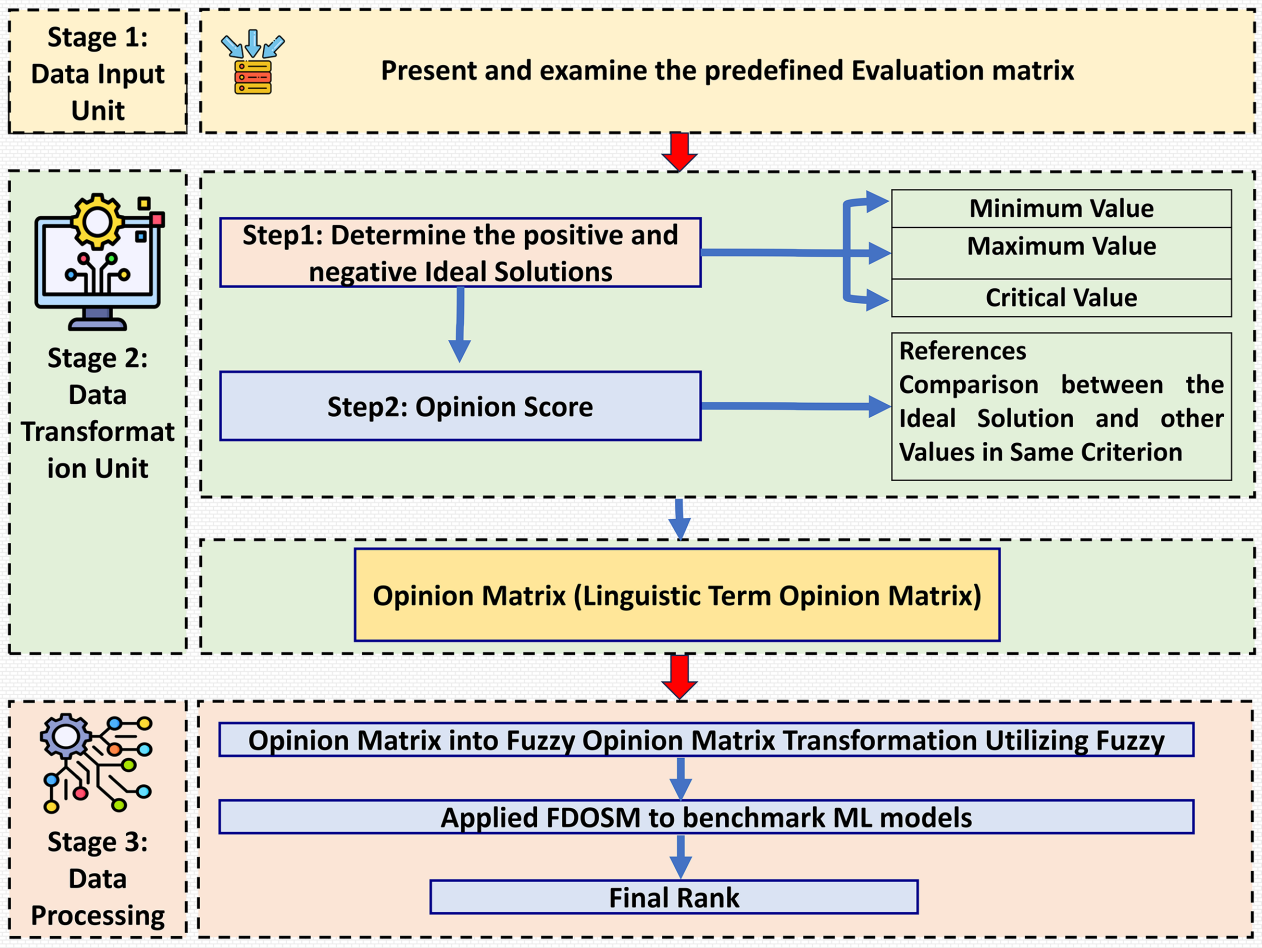
The three-stage FDOSM workflow: (1) data input unit establishes the evaluation matrix and ideal solutions, (2) transformation unit converts values to linguistic opinion scores, and (3) processing unit generates fuzzy rankings for model benchmarking.

This section introduces the decision-making method in which fuzzy logic is used, incorporating subjective opinions and benchmarking ML models. This method is called the FDOSM (Jassim, Abd & Omri, 2023). This mathematical method is used to solve multicriteria decision-making problems with a single decision. Figure 2 shows the three main stages of FDOSM model, which are the data input unit (which implements a decision matrix (DM)), the data transformation unit (which converts the DM into an opinion DM), and the data processing unit (which employs a fuzzy opinion matrix to determine the ranking for each alternative (ML algorithms)).

#### Data input unit

This stage is similar to the MCDM method; in which any decision-making problem has m alternatives (ML models), $A1,A2,A3,\ldots,Am$ and $n$ sets of criteria $C1,C2,C3,\ldots,Cn$. Table 7 shows that the decision matrix (DM) corresponds to the ML models. The next stage converts this DM into an opinion matrix (Liu, Eckert & Earl, 2020).

**Table 7:** Decision matrix for the diagnostic ML models.

Alternatives		Performance evaluation metrics (criteria)						
Diagnosis models		C1	C2	C3	C4	C5	C6	C7
A1	XGBoost	C1-A1	C2-A1	C3-A1	C4-A1	C5-A1	C6-A1	C7-A1
A2	KNN	C1-A2	C2-A2	C3-A2	C4-A2	C5-A2	C6-A2	C7-A2
A3	DT	C1-A3	C2-A3	C3-A3	C4-A3	C5-A3	C6-A3	C7-A3
A4	RF	C1-A4	C2-A4	C3-A4	C4-A4	C5-A4	C6-A4	C7-A4
A5	SVM	C1-A5	C2-A5	C3-A5	C4-A5	C5-A5	C6-A5	C7-A5
A6	ANN	C1-A6	C2-A6	C3-A6	C4-A6	C5-A6	C6-A6	C7-A6
A7	SGD	C1-A7	C2-A7	C3-A7	C4-A7	C5-A7	C6-A7	C7-A7
A8	GBoost	C1-A8	C2-A8	C3-A8	C4-A8	C5-A8	C6-A8	C7-A8
A9	LGBM	C1-A9	C2-A9	C3-A9	C4-A9	C5-A9	C6-A9	C7-A9
A10	AdaBoost	C1-A10	C2-A10	C3-A10	C4-A10	C5-A10	C6-A10	C7-A10
A11	NB	C1-A11	C2-A11	C3-A11	C4-A11	C5-A11	C6-A11	C7-A11
A12	ET	C1-A12	C2-A12	C3-A12	C4-A12	C5-A12	C6-A12	C7-A12

**Note:**

C, Criteria; A, alternative; C1, precision; C2, recall; C3, F1-score; C4, kappa; C5, hamming loss; C6, MCC; C7, accuracy.

#### Data transformation unit

This stage is depends on stage one, which belongs to the DM. The FDOSM method selects the ideal solution with three parameters, which are the Min, Max, and critical values. The lowest value for Min means the better solution, and the highest value for Max means the best solution. In addition, the critical value is used when the ideal solution is neither Min nor Max. This work can select the ideal solution for immeasurable values. The transformation unit consists of two steps to convert the DM into an opinion matrix (OM) to find the ideal solution (Al-Qaysi et al., 2023):

**Step 1:** This step selects the ideal solution for each criterion used in the DM for the diagnosis of rheumatic and autoimmune diseases, as determined by experts (subjects), based on Eq. (12) (Al-Samarraay et al., 2022). A subjective expert with more than 6 years of experience in the ML approach for disease diagnosis was selected.

(12) $${A^*} = \{ [ma{x_{i}}({\upsilon _{ij}}),mi{n_{i}}({\upsilon _{ij}})\quad for\quad j\epsilon J,O{p_{ij}}\epsilon I.J\quad where\quad i = 1,2,3, \ldots ,m]\}$$

where ${\upsilon _{ij}}$ is the value of the cell at the ${i^{th}}$ row and the ${j^{th}}$ column in the DM, as shown in Table 7, $m$ denotes the total number of DMs, max represents the ideal value for the benefit criteria, $min$ represents the ideal value solution for the cost criteria and $O{p_{ij}}$ denotes the critical value is the ideal value between the minimum and maximum values.

**Step 2:** This step describes the reference comparison between the ideal solution and the alternative values for each criterion. Five scales are used to compare the linguistic terms: No Difference (ND), Slight Difference (SD), Difference (D), Big Difference (BD), and Huge Difference (HD). The ideal solution selection step is followed by comparing the ideal solution with the value of alternatives in the same criterion, as shown in Eq. (13). In this work, an expert (judge) asks whether the relevant differences have significantly changed the opinion of the DM.

(13) $$O{P_{Lang}} = \{ (({\upsilon _{ij}})\sim \otimes {\upsilon _{ij}}|j\epsilon J)|i = 1,2,3, \ldots ,m]\}$$

where $\otimes$ represents the reference comparison between the ideal solution and the alternatives as the scale. The final result of this step is an OM obtained from each expert, as shown in Eq. (14).

(14) $$O{P_{Lang}} = \left[ {\matrix{ {{A_{1}}} \cr  \vdots \cr  {{A_{m}}} \cr  } } \right]\left[ {\matrix{ {o{p_{1,1}}} & {o{p_{1,2}}} & \cdots & {o{p_{1,n}}} \cr  {o{p_{2,1}}} & {o{p_{2,2}}} & \cdots & {o{p_{2,n}}} \cr  \vdots & \vdots & \ddots & \vdots \cr  {o{p_{m,1}}} & {o{p_{m,2}}} & \cdots & {o{p_{m,n}}} \cr  } } \right]. $$

Once the OM has been formulated, the next step is to convert it into fuzzy numbers *via* suitable fuzzy membership functions. This conversion process helps quantify the linguistic terms expressed in the OM and represents them as fuzzy numbers, which provide a more precise and quantitative representation of the experts (judgments).

#### Data processing unit

The OM refers to the output of the transformation unit (Albahri et al., 2024). The final stage begins by transferring the OP to a fuzzy opinion DM by converting the linguistic terms of the OM into fuzzy numbers. This stage begins by transforming the OM into a fuzzy opinion *via* triangular fuzzy members (TFM). The TFMs shown in Table 8 are used to replace the linguistic terms obtained from the experts on the basis of three values for TFM; these values are ${V_{1}},{V_{2}},and\;{V_{3}}$. These values are replaced by TFMs, which are defined by their membership function. Equation (15) is used for the fuzzy score. Equation (16) presents the rank for all the ML models. In this work, the lowest value corresponds to the best ML model.

(15) $$S(A) = ({V_{1}} + {V_{2}} + {V_{3}})/3$$

(16) $${R_{i}} = 1 + \sum\nolimits_{j = 1}^N \; 1({S_{j}}\lt{S_{i}}).$$

**Table 8:** Linguistic terms and their corresponding TFM values.

Linguistic terms	TFM		
	$V_{1}$	$V_{2}$	$V_{3}$
ND	0.0	0.10	0.30
SD	0.10	0.30	0.50
D	0.30	0.50	0.75
BD	0.50	0.75	0.90
HD	0.75	0.90	1.00

To illustrate the process, suppose that we have three ML models (SVM = A1, KNN = A2, and DT = A3) and two criteria (precision and accuracy).

**Step 1:** DM construct (using Table 7)

Create a DM where the rows represent the alternatives (models) and the columns represent the criteria (evaluation metrics), as shown in Table 9.

**Table 9:** Example using FDOSM for three models and two criteria.

Model	Precession	Accuracy
A1	75.1	74.5
A2	72	71.3
A3	83.6	82.9

**Step 2:** Determine the ideal solution (using Eq. (12))

For each criterion, identify the ideal value, max for benefit criteria. for max precision DT = 83.6 and accuracy also DT = 82.9.

**Step 3:** Transform DM into OM

An expert compares each alternative’s value to the ideal solution using Eq. (14) then using linguistic terms in Table 8.

For A1, the precision range is (75.1–83.6), which corresponds to the linguistic term D, and the accuracy range is (74.3–82.9), also classified as D. For A2, the precision range is (72.0–83.6), corresponding to BD, and the accuracy range is (71.3–82.9), also mapped to BD. Finally, A3 has both precision and accuracy fixed at (83.6–83.6) and (82.9–82.9), respectively, which are both categorized as ND.

**Step 4:** Convert linguistic terms to fuzzy numbers (using Table 8)

Replace all terms with TFM to value, for ND = (0.0, 0.1, 0.3), D = (0.3, 0.5, 0.75), and BD = (0.5, 0.75, 0.9).

**Step 5:** Calculate fuzzy scores (using Eq. (15))

For each alternative, compute the average of fuzzy numbers. For A1 = 1.0334, A2 = 1.4334, and A3 = 0.2666.

**Step 6:** Calculate fuzzy scores (using Eq. (16))

Lower scores indicate better performance. A1 = 1.0334 (Rank 2), A2 = 1.4334 (Rank 3), and A3 = 0.2666 (Rank 1) which is the best algorithm.

## Results and discussion

This section provides an analysis of the findings of the experiments and evaluations conducted during this work. It is mainly concerned with the results achieved from predicting different diseases *via* applied ML models, FDOSM methods, and explainable artificial intelligence techniques. Simplified and illustrated representations of data, such as those provided by principal component analysis (PCA), is the interesting part, particularly owing to how they influence classification accuracy. The discussion is not only presents the key results but also enables concerns such as class weights, class overlap occupancy classes and features and their selection with the need to move to different ways to be regarded in detail to improve diagnosis and decision making in medical applications.

### Dataset analysis

Various clinical features revealed several important aspects, including count and missing data, with descriptive statistics presented in Table 10. The age and gender features are complete without any missing values. Continuous characteristics, such as ESR, CRP, RF or anti-CCP, suffer deficits in values of more than 9% to 27%. Binary features such as HLA-B27, ANA, Anti-Ro, and Anti-La are also features with missing value proportions between 16% and 43%. On the other hand, there are clinical features such as Anti-dsDNA and Anti-Sm that have 43% missing values, which could pose challenges in modeling. Finally, C4 and C3, which have moderate missing values between 14% and 17%. Addressing the missing values, appropriately normalizing the continuous variables, and adjusting binary variables will be necessary to obtain proper and exact modeling, especially since many features are missing for some of the variables.

**Table 10:** Descriptive statistics and data completeness for demographic and clinical features.

Feature	Count	Missing/Percentage (%)	Min/max	Mean/Std
Age	12,085	0/0	20/80	29.905/17.649
Gender	M(6,151)/F(5,934)	0/0	–	0.508/0.499
ESR	10,997	1,088/9	0.0001/49.99	24.698/14.38
CRP	9,668	2,417/20	0.1018/29.99	13.299/10.37
RF	10,756	1,329/11	0.0043/39.99	19.691/11.51
Anti-CCP	8,822	3,263/27	0.0002/39.99	19.755/11.58
HLA-B27	P(6,217)/N(3,934)	1,934/16	–	0.635/0.482
ANA	P(5,294)/N(3,045)	3,746/31	–	0.6124/0.487
Anti-Ro	P(5,305)/N(3,880)	2,900/24	–	0.6348/0.481
Anti-La	P(5,354)/N(3,710)	3,021/25	–	0.577/0.493
Anti-dsDNA	P(4,083)/N(3,289)	4,713/39	–	0.59/0.491
Anti-Sm	P(3,734)/N(3,154)	5,197/43	–	0.553/0.497
C3	10,393	1,692/14	50/205.94	132.43/36.28
C4	10,031	2,054/17	5/74.98	38.80/20.06

**Figure 3:**
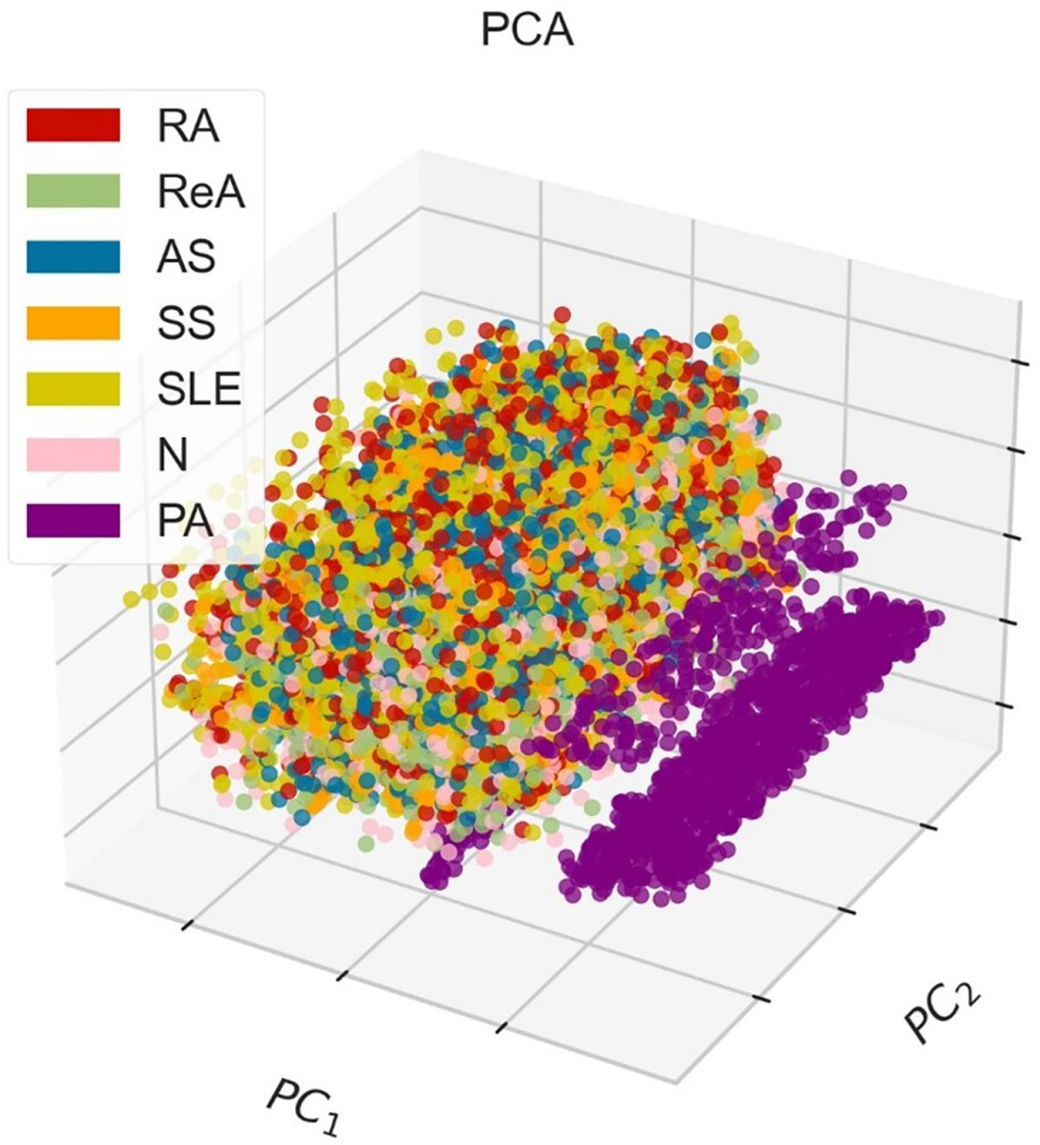
3D-PCA visualization of the dataset, where PC1 (52.49%) and PC2 (13.68%) collectively explain 66.17% of the total variance, demonstrating moderate class separability for rheumatic diseases (*e.g*., RA, SLE) but overlap for AS and ReA due to shared inflammatory markers.

Figure 3 shows the PCA method in 3D, where points that indicate instances of different classes, corresponding to various diseases. The demonstrative graph represents a visual projection of the dataset on the first two (PC1, PC2), where the PA class appears to be isolated, whereas other pathological classes appear to blend in the middle to a large extent. This blend suggests that there may be some overlap in the features associated with these diseases and that additional or more advanced discrimination methods might be necessary to fully separate them.

### Machine learning results

This section provides performance metrics in terms of precision, recall, F-score, kappa, Hamming loss, MCC, accuracy, and other metrics for several ML models for unbalanced datasets and balanced datasets. Table 11 shows the unbalanced dataset, indicating that GBoost, RF, XGBoost, ET, and the LGBM perform better. While, GBoost and the RF outperforming many other metrics exhibiting high precision and well-balanced F-scores with low Hamming loss, exceptional MCC, and kappa scores reflecting high prediction reliability. Others, such as DT, ANN, and SGD, are in the middle tier of the models with an average performance level. However, SVM scores better on the reliability scale and poorer on the accuracy scale. In terms of KNN and NB, the two methods performed poorly, especially in terms of Hamming loss and lower accuracy. AdaBoost is the outlier, which performs hopelessly across all the metrics. In general, the ensemble models performed the best concerning this unbalanced dataset, whereas poorer performing models such as KNN, and NB, while AdaBoost did not perform as well.

**Table 11:** Performance comparison of ML models on an unbalanced dataset.

Model	Precision	Recall	F-score	Kappa	Hamming loss	MCC	Accuracy
XGBoost	83.193	81.034	81.855	78.692	17.761	78.733	82.239
KNN	62.539	61.986	61.776	58.851	34.28	58.966	65.72
DT	78.864	78.769	78.782	75.554	20.436	75.564	79.564
RF	86.025	80.513	82.278	80.168	16.464	80.291	83.536
SVM	62.785	64.669	63.48	65.729	28.351	65.957	71.649
ANN	78.01	76.805	77.344	75.563	20.381	75.571	79.619
SGD	71.887	71.157	68.581	66.583	27.91	67.8	72.09
GBoost	86.544	82.036	83.543	80.665	16.078	80.78	83.922
LGBM	83.449	81.445	82.216	79.122	17.402	79.165	82.598
AdaBoost	21.98	38.78	25.173	24.721	62.355	28.626	37.645
NB	70.559	60.116	57.004	50.41	41.975	52.978	58.025
ET	83.014	76.535	77.98	77.462	18.698	77.591	81.302

**Figure 4:**
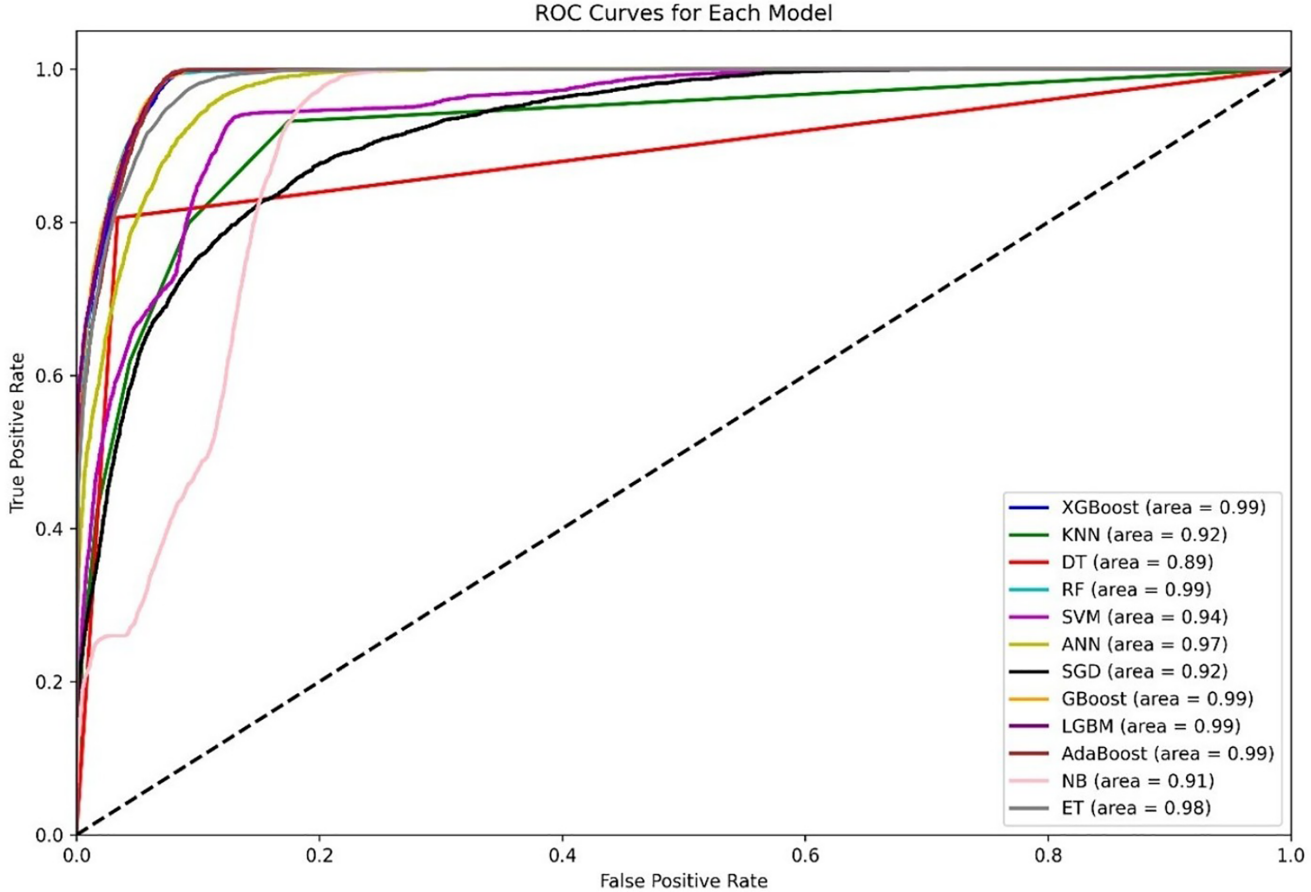
Comparative ROC curves of 12 ML models using an unbalanced dataset, with ensemble methods (XGBoost, GBoost, RF, LGBM, and AdaBoost) achieving near-perfect AUC (¿0.99), outperforming simpler models like DT (AUC = 0.89).

Figure 4 shows a diagram of the ROC curve that enables us to perform a comparative assessment of the capabilities of different ML models that are based on distinguishing among classes. This evaluation takes the AUC results in the form of calculating the area under the curve. XGBoost, GBoost, AdaBoost, RF, and LGBM exhibit superior performance classifiably, with corresponding AUCs of approximately 0.99, whereas ET, ANN, and SVM also perform well, with AUCs of 98, 0.97, and 0.94, respectively. Conversely, KNN and SGD delivers the AUC of 0.92, this may be due to their imperviousness to the problem of high-dimensional data. Most apparently, NB, despite its simplifying assumption, records an efficient AUC of 0.91.

For the AUC measure, the DT performs lowest, with an AUC of 0.89, which is a likely overfitting metric. Additionally, all boosting algorithms appear to be more efficient than DT because of easier interactions of noise data. Some modifications in terms of feature scaling such as feature selection to reduce overfitting.

Table 12 outlines the evaluation measures of different combinations of ML models and highlights the level of overfitting. Each model in this case, XGBoost, DT, RF, LGBM, ET, and GBoost, showed overfitting tendencies to varying degrees, and this performance gap between training and validation scores was large, with DT recording of 20.24% drop in training performance. Such models cannot cope with overfitting without adopting regularization or pruning. In contrast, SVM, NB, AdaBoost, and SGD have no overfitting, and all training and validation scores are similar for such models, with some, such as AdaBoost and NB, being less effective in terms of performance. The overall performance of the ANN is balanced, whereby both training and validation do not perform outliers centered on the degree of overfitting that exists. Instead, tree-based models usually require tuning to overcome low performance, while tuning is likely to increase generalization. Further, whereby low-performing models may even are optimized or other measures taken without creating the risk of incorporating generalization into the validity. In this test, we can use Eq. (17) to calculate the percentage if overfitting occurs. It uses the difference between training and validation.

(17) $$Overf = \left( {{{Train\mbox{-}Validation} \over {Train}}} \right)*100.$$

**Table 12:** Training and validation for all ML models with cross-validation K = 5.

Model	Train scores mean	Validation scores mean	Overfitting	
			Score%	Status
XGBoost	0.9998	0.8295	17.03%	Yes
KNN	0.7943	0.6883	13.36%	Mild
DT	1	0.7976	20.24%	Yes
RF	1	0.8337	16.63%	Yes
SVM	0.7244	0.7214	0.41%	No
ANN	0.7945	0.7749	2.47%	Mild
SGD	0.6353	0.6359	-0.09%	No
GBoost	0.9174	0.841	8.33%	Yes
LGBM	1	0.8334	16.66%	Yes
AdaBoost	0.3576	0.3524	1.46%	No
NB	0.59	0.5887	0.22%	No
ET	1	0.8127	18.73%	Yes

Table 12 shows three overfitting statuses: yes, no, and mild. In the term yes, we have significant overfitting (overfitting % $\ge$ 10%), whereas mild refers to moderate overfitting (5% $\le$ Overfitting % < 10%). Finally, no refers to no overfitting (overfitting percentage < 5%).

Table 13 shows that the analysis of several ML models in a balanced dataset confirms that in models such as XGBoost, RF, GBoost, LGBM, and ET, the other models outperform in terms of all the metrics. XGBoost yields outstanding results, with the accuracy of 86.131%. Among the methods evaluated, GBoost was ranked as the best performing model, with 86.889% accuracy, indicating its strength. Moreover, linear models such as SGD, NB, and AdaBoost do not perform adequately, and with AdaBoost, the worst performance reflects accuracy of 34.397%. Overall, the performance of the ensemble models, especially GBoost, is superior and reliable; however, all the other models have unpredictable outcomes, and the performance is very poor because the balanced dataset is not favorable, particularly for the linear model AdaBoost. Note that after our dataset is balanced, the size of each class becomes ReA (2,946), RA (2,848), AS (2,127), SS (1,852), PA (1,783), N (1,604), and SLE (1,355).

**Table 13:** Performance metrics of various ML models on a balanced dataset.

Model	Precision	Recall	F-score	Kappa	Hamming loss	MCC	Accuracy
XGBoost	86.497	86.226	86.313	83.628	13.869	83.647	86.131
KNN	72.007	71.093	70.653	65.95	28.726	66.456	71.274
DT	83.649	83.515	83.574	79.875	17.061	79.878	82.939
RF	86.228	85.563	85.595	83.059	14.328	83.177	85.672
SVM	75.118	73.77	73.26	69.478	25.741	69.984	74.259
ANN	83.368	82.166	82.268	78.433	18.232	78.726	81.768
SGD	74.089	69.201	65.94	62.855	31.206	64.452	68.794
GBoost	87.347	86.78	86.802	84.511	13.111	84.624	86.889
LGBM	86.204	86.01	86.035	83.387	14.076	83.414	85.924
AdaBoost	36.71	33.099	21.684	21.255	65.603	26.015	34.397
NB	75.45	64.953	61.846	56.775	37.015	58.783	62.985
ET	86.309	85.309	85.634	82.689	14.65	82.73	85.35

**Figure 5:**
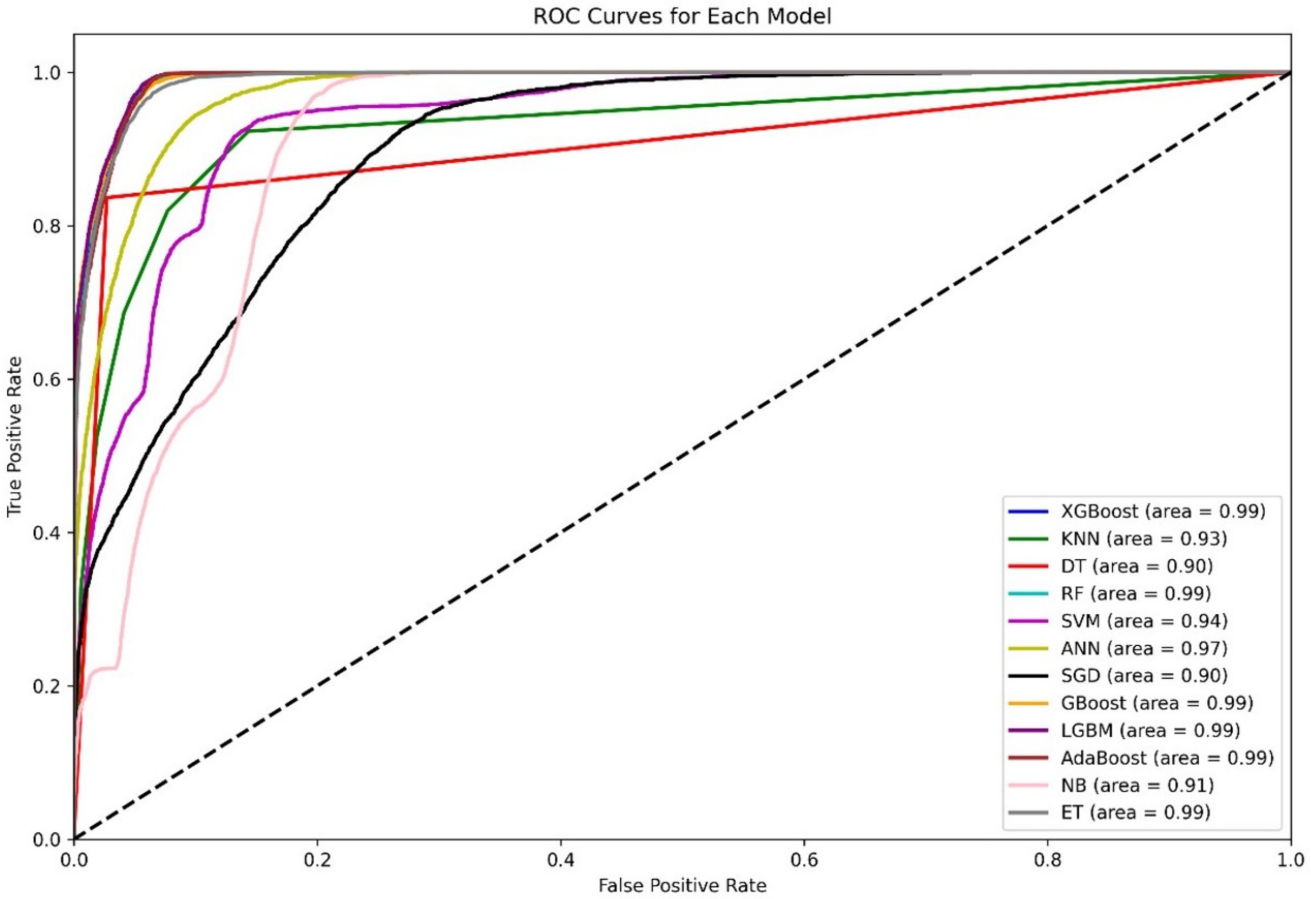
ROC curves for 12 ML models using the balanced dataset.

The ROC curves in Fig. 5 clearly show that other ensemble models, such as XGBoost, RF, Gboost, AdaBoost, and LGBM, are the clear winners, as shown by their almost perfect AUC values, which indicates that such models can learn complex patterns and generalize well on various datasets. ANN performance is classifiable, with a corresponding AUC of 97, whereas SVM and KNN also perform well, with AUCs of 0.94 and 0.93, respectively. Most apparently, NB, despite its simplifying assumption, records an efficient AUC of 0.91. Conversely, KNN and SGD deliver an AUC of 0.90, this may be due to their imperviousness to the problem of high-dimensional data.

From the information in Table 14, the overfitting in various models is compared on the basis of their training and validation performance. XGBoost, DT, RF, LGBM, and ET exhibit heavy overfitting since they attain pleasant training scores, while their validation scores roll down and roll down a high margin, indicating that training is aimed at memorizing certain patterns and there are few opportunities to expose the models to new data. The KNN and gradient boosting models show moderate overfitting, meaning that for these methods, the overall performance is reasonable, although it could be increased with tuning or additional datasets to avoid overfitting. The SVM, ANN, SGD, AdaBoost, and NB methods were delayed or had no overfitting since the training and validation scores were reasonable.

**Table 14:** Training and validation for all ML models with cross-validation K = 5.

Model	Train scores mean	Validation scores mean	Overfitting	
			Score%	Status
XGBoost	0.9999	0.8542	14.6%	Yes
KNN	0.8035	0.7167	10.8%	Mild
DT	1.0	0.8218	17.8%	Yes
RF	1.0	0.861	13.9%	Yes
SVM	0.7478	0.7417	0.8%	No
ANN	0.8144	0.7999	1.8%	No
SGD	0.6207	0.618	0.4%	No
GBoost	0.9237	0.8628	6.6%	Mild
LGBM	1.0	0.861	13.9%	Yes
AdaBoost	0.3931	0.3933	−0.05%	No
NB	0.6395	0.6386	0.1%	No
ET	1.0	0.852	14.8%	Yes

### FDOSM results

In this stage, three experts working with ML and evaluation were used. The experts have more than 6 years in this field. Tables 15, 16, and 17 present evaluations from three different experts on various machine learning models on the basis of multiple performance metrics.

**Table 15:** Expert one evaluation of 12 ML models using linguistic terms.

Model	Precision	Recall	F-score	Kappa	Hamming loss	MCC	Accuracy
XGBoost	ND	SD	SD	SD	ND	SD	ND
KNN	BD	BD	D	BD	D	BD	BD
DT	SD	D	SD	SD	ND	SD	SD
RF	ND	SD	ND	ND	ND	ND	ND
SVM	BD	BD	D	D	SD	D	D
ANN	SD	BD	SD	SD	ND	SD	SD
SGD	D	D	D	D	SD	D	D
GBoost	ND	ND	ND	ND	ND	ND	ND
LGBM	ND	SD	ND	SD	ND	SD	ND
AdaBoost	HD	HD	HD	HD	HD	HD	HD
NB	D	BD	BD	BD	BD	BD	BD
ET	ND	D	SD	SD	ND	SD	ND

**Table 16:** Expert two evaluation of 12 ML models using linguistic terms.

Model	Precision	Recall	F-score	Kappa	Hamming loss	MCC	Accuracy
XGBoost	ND	ND	ND	SD	ND	SD	ND
KNN	D	D	D	BD	D	BD	D
DT	SD	ND	SD	SD	ND	SD	SD
RF	ND	SD	ND	ND	ND	ND	ND
SVM	D	D	D	D	SD	D	SD
ANN	SD	SD	SD	SD	ND	SD	SD
SGD	SD	SD	D	D	SD	D	SD
GBoost	ND	ND	ND	ND	ND	ND	ND
LGBM	SD	ND	ND	SD	SD	ND	ND
AdaBoost	HD	HD	HD	HD	HD	HD	HD
NB	SD	D	BD	BD	BD	BD	BD
ET	ND	SD	SD	SD	ND	SD	ND

**Table 17:** Expert three evaluation of 12 ML models using linguistic terms.

Model	Precision	Recall	F-score	Kappa	Hamming loss	MCC	Accuracy
XGBoost	ND	ND	SD	SD	ND	SD	ND
KNN	BD	BD	BD	BD	D	BD	BD
DT	SD	SD	SD	SD	ND	SD	SD
RF	ND	SD	ND	ND	ND	ND	ND
SVM	D	BD	BD	D	SD	D	SD
ANN	D	SD	SD	SD	ND	SD	SD
SGD	SD	D	D	D	SD	D	SD
GBoost	ND	ND	ND	ND	ND	ND	ND
LGBM	SD	ND	ND	ND	ND	SD	ND
AdaBoost	HD	HD	HD	HD	HD	HD	HD
NB	D	D	BD	BD	BD	BD	BD
ET	ND	SD	SD	SD	ND	SD	ND

Table 18 shows the benchmarking performance scores and the classifications by different ML models by three experts and a final aggregated consensus decision. The final decision column incorporates all the factors and includes the corresponding aggregate score and rank for each model. In the case of GBoost, the best among all the models with all three experts ranked 1st, with a score of 0.1333, which demonstrates perfect compliance with the evaluations. Additionally, the RF performs well and always holds second place, with a score of 0.1571 in each expert’s evaluation, indicating that there is no disagreement about how well it performs. LGBM has been placed in 3rd place in the aggregated score. XGBoost occupies with the final score of 0.2048 and ranks 4th, implying an average performance. In contrast, AdaBoost emerged as the least rated model 12th by all the experts, with a lower scaling of 0.8833; hence, it is the least efficient model assumed.

**Table 18:** Model evaluation by experts and final decision rankings.

Model	Expert one		Expert two		Expert three		Final decision	
	Score	Rank	Score	Rank	Score	Rank	Score	Rank
XGBoost	0.2286	4	0.181	3	0.2048	4	0.2048	4
KNN	0.6595	10	0.5738	10	0.6881	11	0.6405	10
DT	0.3071	6	0.2524	6	0.2762	6	0.2786	6
RF	0.1571	2	0.1571	2	0.1571	2	0.1571	2
SVM	0.5429	9	0.4548	9	0.5119	9	0.5032	9
ANN	0.3357	7	0.2762	7	0.3071	7	0.3063	7
SGD	0.4857	8	0.3929	8	0.4238	8	0.4341	8
GBoost	0.1333	1	0.1333	1	0.1333	1	0.1333	1
LGBM	0.2048	3	0.2048	4	0.181	3	0.1969	3
AdaBoost	0.8833	12	0.8833	12	0.8833	12	0.8833	12
NB	0.6881	11	0.6286	11	0.6595	10	0.6587	11
ET	0.2595	5	0.2286	5	0.2286	5	0.2389	5

**Figure 6:**
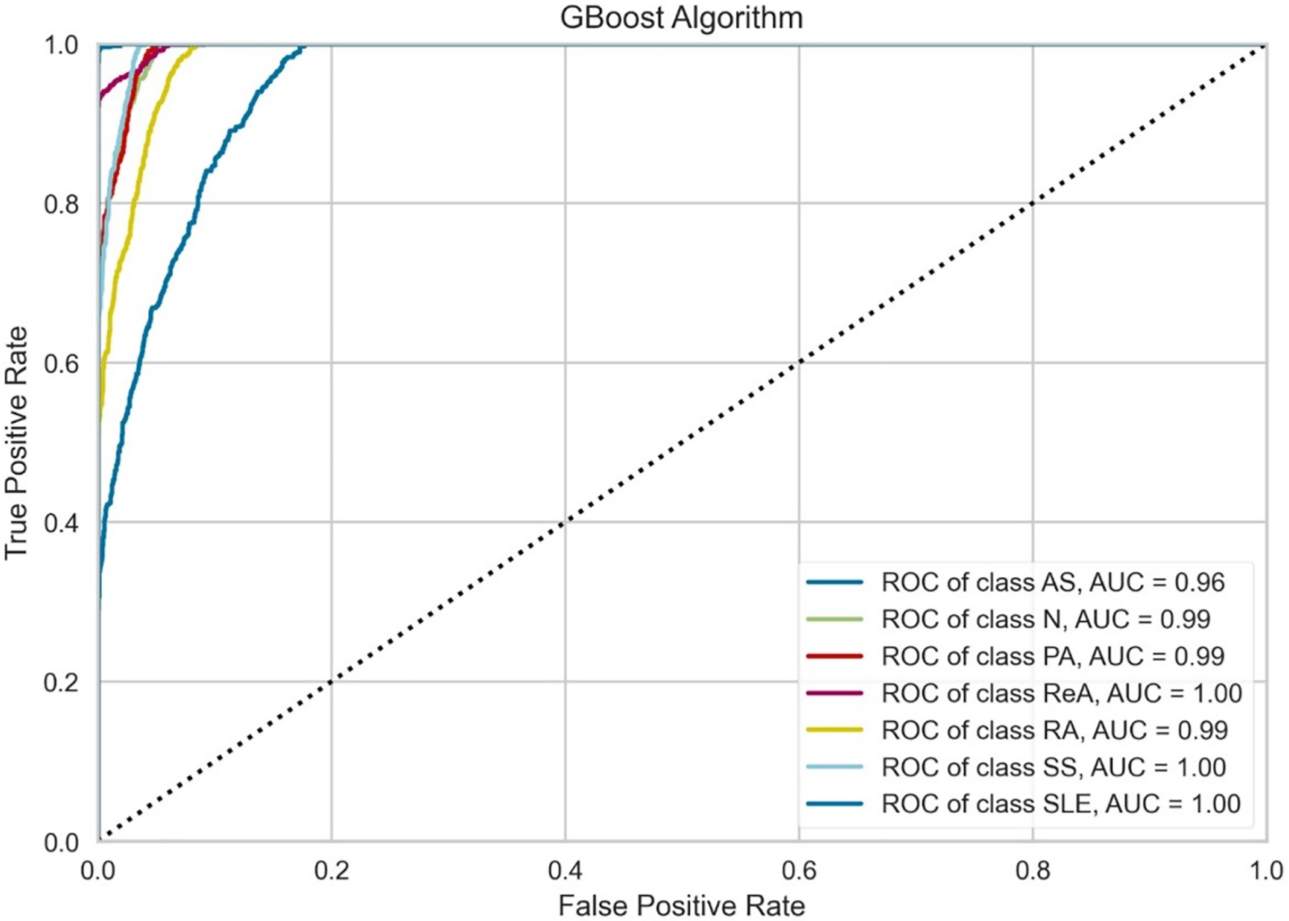
Class-wise ROC curves for the GBoost model. Each curve represents the true positive rate *vs* the false positive rate for a specific class, with the AUC indicating the discriminative performance of the model for that class.

Figure 6 indicates that the performance of the GBoost algorithm exceeds that of the other algorithms with respect to the AUC values. In addition, the model performs perfect classification in a number of classes, which means that it is well optimized for the task and it is capable of determining the majority of these diseases. The grade of performance for AS is slightly lowers with highly accurate. The slight proximity of the curves to the lower zones of false positive rates suggests that there could be some problems in the early detection stage or in cases where some of the signs presented by one of the diseases are also exhibited by another illness. However, the results prove that there is a place for the GBoost algorithm with respect to the classification of these diseases, as it strongly varies in the diagnosis of ReA, SS, and SLE and differentiates healthy (normal) people from patients suffering from these autoimmune conditions.

**Figure 7:**
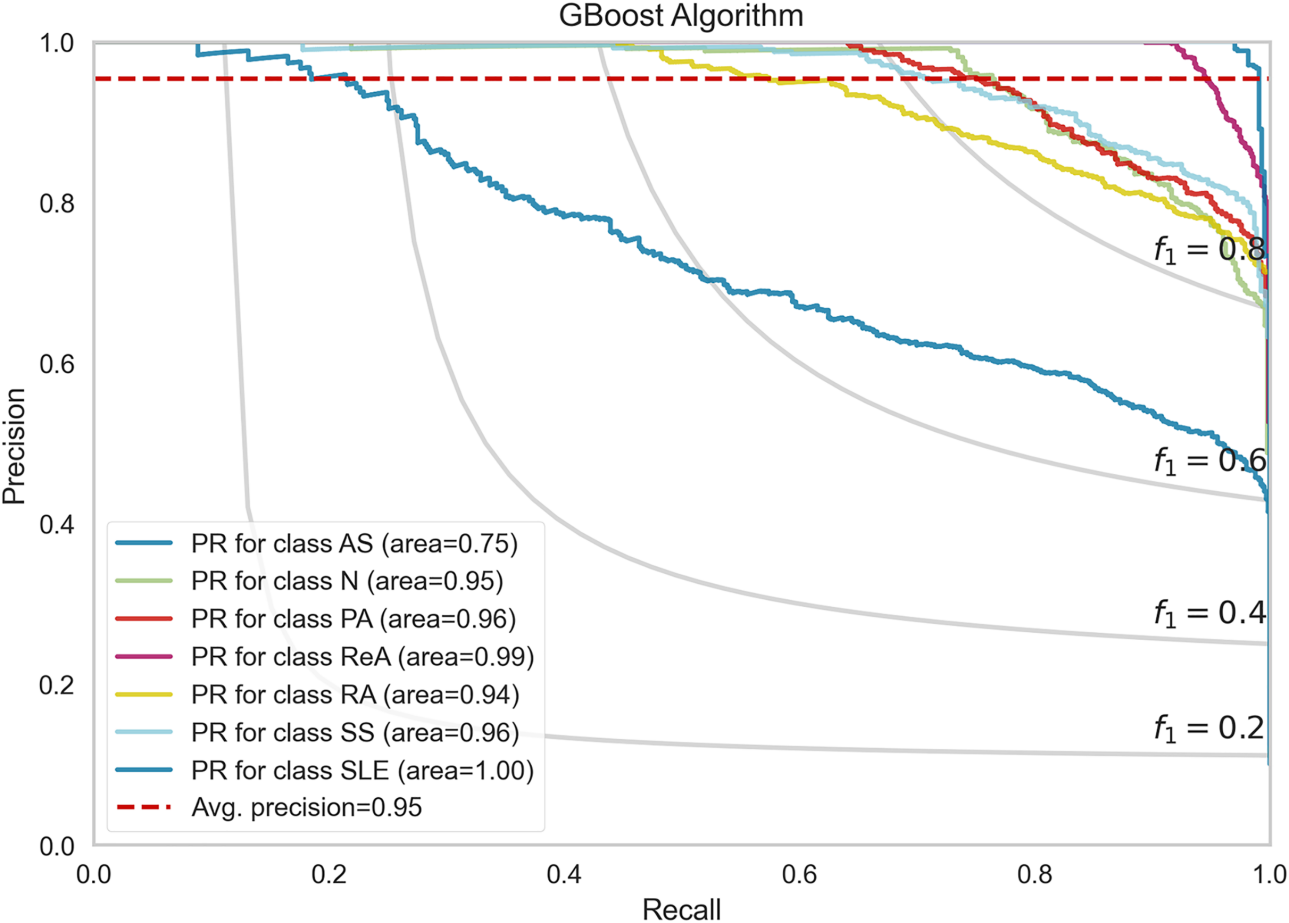
Precision-recall trade-off per disease class, highlighting GBoost’s strongest performance for SLE (F1 = 1.0) and challenges with AS (F1 = 0.89) from shared clinical markers.

Figure 7 shows the performance of the precision–recall (PR) curves of the GBoost algorithm in classes of rheumatic and autoimmune diseases. The accuracy or the PR score is helpful for skewed datasets, as it considers the relationship between precision and recall. The mean average precision for the entire GBoost algorithm is 0.95, which means that the model can dispose of positive predictions across most classes while making most positive predictions correct across all classes. The plot also includes lines of F1-scores, which illustrate the dependence of precision and recall on the threshold. The PR of SLE is 1.00, which essentially means that the disease diagnosis is perfect and has both recall and precision of nearly unity the entire time, that is, no false allegations or misses. The GBoost algorithm in the majority of classes provides good prediction of the majority of diseases with high precision and high recall, with the exception of AS, and this condition is likely related to symptoms of other diseases.

### Evaluation analysis

This section focuses on two important key aspects, XAI and comparative analysis, to evaluate our framework. These keys show that our framework not only delivers accurate predictions but also provides transparency and interpretability, alongside a comprehensive comparison of various machine learning techniques.

#### XAI

XAI is an area of work that has rapidly expanded in recent years and focuses on improving the interpretability of ML models. The main objective of XAI is to design models in such a way that the reasons for its outputs can be clarified to the human being interfaced with the system. This can involve providing reasons for individual predictions or explaining why a model prediction is made in general and/or what features are relevant to the model’s outputs. In this work, use Local interpretable model-agnostic explanations (LIME) are a tool that makes ML models more understandable by providing default explanations of the decisions made by them.

In this work, three case studies were used to demonstrate the superiority and rationality of the proposed model. In Table 19, the following clinical characteristics are provided for the correct diagnosis of patients: ReA (case 1), PA (case 2), and N (case 3). In general, the characteristic weights reveal that there are different and sometimes conflicting roles of clinical markers in differentiating the pathological states of patients, particularly inflammatory markers and autoantibodies in certain cases, whereas they are less important in others.

**Table 19:** Three cases from the testing set.

Feature	Case 1		Case 2		Case 3	
	Weight	Value	Weight	Value	Weight	Value
Age	0.0047	66	0.0054	71	−0.0017	25
Gender	−0.0161	1	−0.0160	1	0.0169	0
ESR	−0.0214	24.003	−0.0691	33.538	0.1527	4.494
CRP	−0.0282	13.539	−0.0154	25.645	0.0025	13.299
RF	0.0506	14.674	0.0404	3.118	0.0448	10.020
Anti-CCP	0.0388	6.449	0.0388	13.132	0.0422	2.257
HLA-B27	−0.0243	1	−0.0250	1	−0.0228	1
ANA	−0.0669	1	−0.0701	1	−0.0723	1
Anti-Ro	−0.0331	0	0.0335	0	−0.0355	1
Anti-La	−0.0302	1	−0.0324	1	−0.0332	1
Anti-dsDNA	0.0166	0	−0.0148	1	0.0149	0
Anti-Sm	−0.0061	1	0.0049	0	−0.0031	1
C3	−0.0329	111.332	−0.0329	107.243	0.0173	147.178
C4	0.0041	39.314	0.0050	64.989	0.0052	38.807
True class	**ReA**		**PA**		**N**	

**Note:**

For gender feature, Female will encoded as 0 and Male will be as 1. For the characteristics (HLA-B27, ANA, Anti-Ro, Anti-La, Anti-dsDNA and Anti-Sm) will Encode (Negative = 0 and Positive = 1).

**Figure 8:**
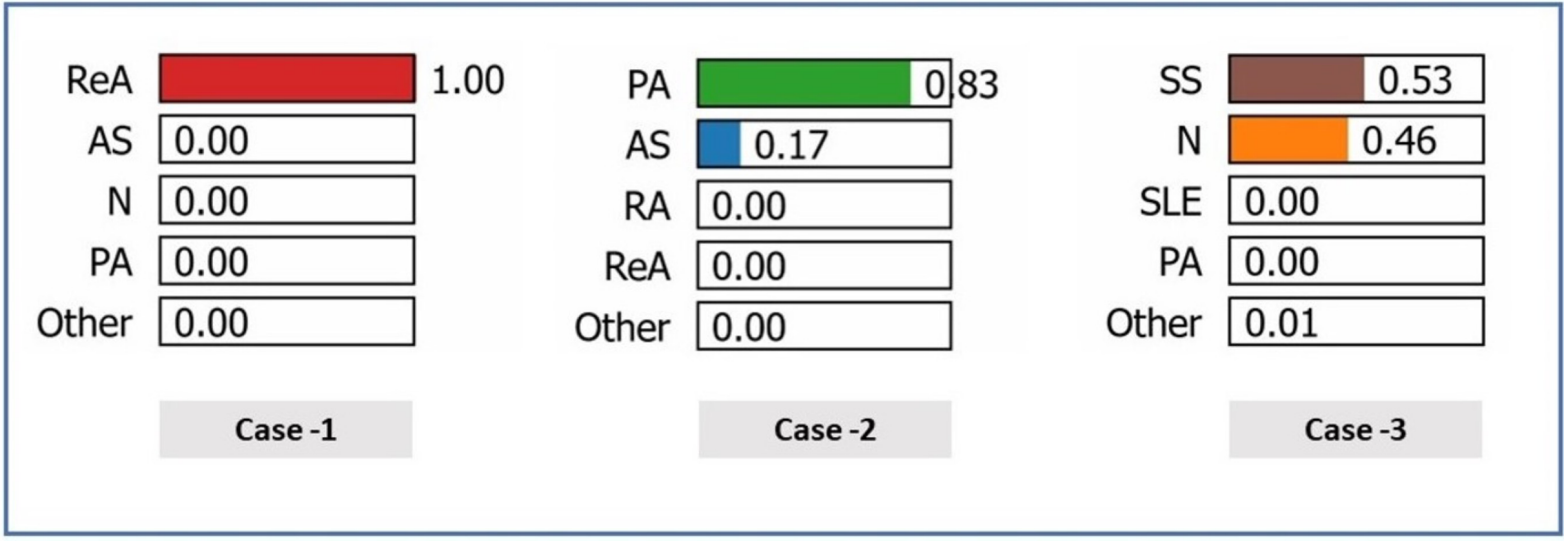
Prediction probabilities for three cases.

Figure 8 shows the predicted probabilities of different diagnostic possibilities for a given medical case *via* the three exploratory diagnostic. In Case 1, the model predicts ReA with 100% confidence, ruling out AS, N, PA, *etc*., all of which are 0.00. The development of probabilities in Case 2 indicates that (83%) is the most likely diagnosis of PA, reducing the chances of AS to a further low (17%) and completely ruling out other states. In Case 3, the model has a mean prediction of (53%) SS and (46%) N. Overall, Case 1 produced the highest level of diagnostic accuracy, Case 2 the second highest, and Case 3 the broadest range of diagnostic potential.

**Figure 9:**
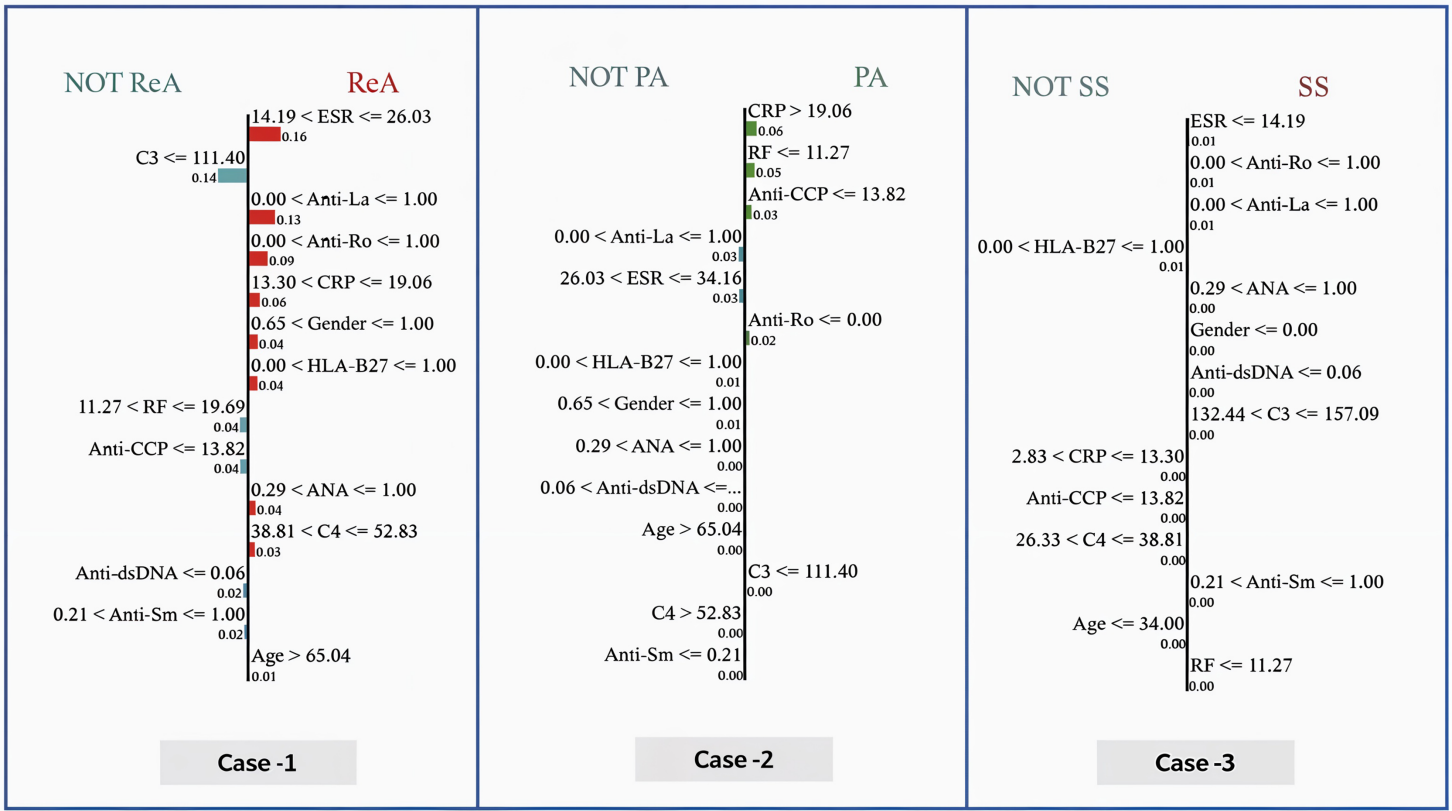
Local interpretable model explanations LIME showing key decision thresholds for disease classification: (Case-1) ReA diagnosis driven by low C3 (111.40 mg/dL) and moderate RF (11.27–19.69 IU/mL), (Case-2) PA prediction requiring high CRP (¿19.06 mg/dL) with negative Anti-Ro, and (Case-3) SS classification dependent on low ESR (14.19 mm/hr) with positive Anti-Ro/La antibodies. Values represent feature contribution weights.

Figure 9 shows the determination of the diagnosis in three cases *via* the GBoost algorithm based on clinical markers, where each of the three cases has a different threshold for one or more of the features as follows:

– In case 1 (ReA), the model predetermines the ESR as an important parameter. When the ESR is less than 26.03, the model assumes lower values of CRP, indicating moderate inflammation. Furthermore, the absence of RF, Anti-CCP, Anti-dsDNA, and Anti-sm, for example, increases the strength of the model in diagnosing ReA. The importance of these features is illustrated by the size of the bars corresponding to them, that is, how much each is involve into the decision.
– In case 2 (PA), the model differentiates patients above the CRP value of 19.06, where more inflammation is likely to be present in the PA. This value is also below the RF index of 11.27 and reflects the blue presence of Anti-CCP. These indicators, when added to the exclusion of HLA-B27 and other biomarkers of autoimmune diseases, are reasonable for the model to consider PA as the most likely diagnosis.
– In case (SS), the model prioritizes the use of an ESR as one of the first steps, considering the erythrocyte sedimentation rate values to be of maximum $\le$14.19 as a very good predictor. Positive findings for anti-Ro and anti-La antibodies push the diagnosis even further toward SS. Lower CRP values and high C3 levels help reduce the number of diagnoses and exclude others.

**Figure 10:**
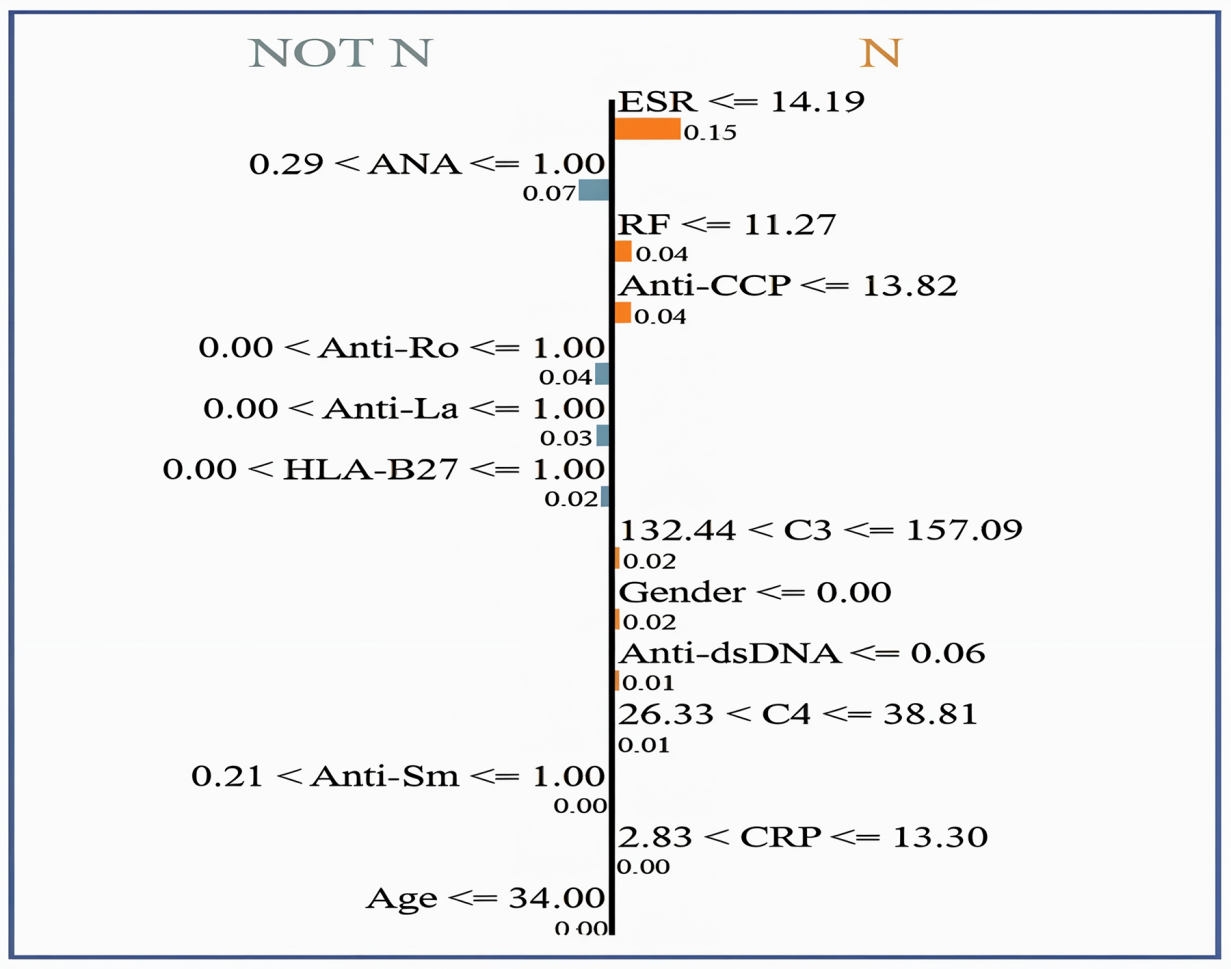
LIME for normal *vs*. non-normal classification, highlighting key decision thresholds. Dominant features favoring a ‘Normal’ classification include low ESR (14.19 mm/hr; weight = 0.15) and negative ANA (1.00; weight = 0.07), with supporting contributions from intermediate C3 levels (132.44–157.09 mg/dL; weight = 0.02) and female sex (weight = 0.02). Values represent normalized feature importance scores from the GBoost model.

Figure 10 shows the interpretability of GBoost algorithm concerning the classification of patients in (N or Not N), which is based on the clinical factors in use. Among Fig. 9 showed features, the most satisfactory feature for pushing the classification toward N is the ESR, as low ESR scores $\le$14.19 help improve the likelihood of a normal report, as evidenced by a relatively high importance score of 0.15. Additionally, reasonably important features are the factors RF and Anti-CCP, with cutoff values of $\le$11.27 and $\le$13.82, respectively. A normal diagnostic status is also supported by C3 levels within the range of 132.44 to 157.09, which score 0.02 in importance. However, the patient’s sex itself made a slight contribution to the *N* classification, with a score of 0.02. Anti-dsDNA levels $\le$0.06 are also a long way from promoting normalcy. C4 levels $\le$38.81 favor an *N* diagnosis with a very subtle importance score of 0.01. In contrast, high levels of ANA (>1.00) greatly help the classification of Not *N* so that it has an importance score of 0.07, which denotes an abnormal condition and explains why it is very useful.

**Figure 11:**
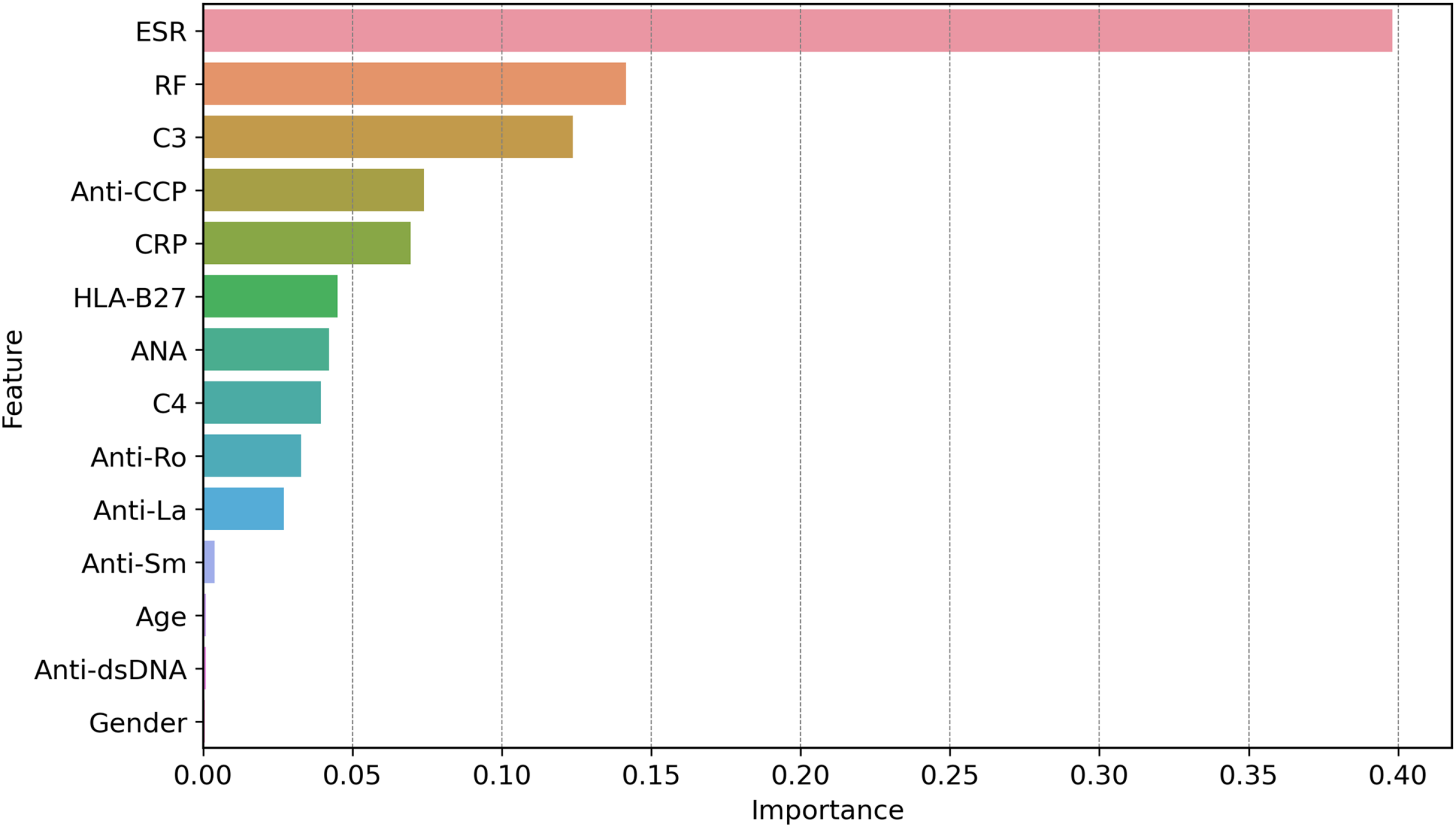
Global feature importance *via* XAI, revealing ESR (score = 0.40) and RF (0.35) as top diagnostic markers, aligning with clinical relevance in autoimmune inflammation.

Figure 11 shows how globally XAI important features are for the prediction of autoimmune and rheumatic diseases. The ESR is the most important factor, with an importance score of approximately 0.40, due to its close relationship with inflammation in autoimmune diseases. The second feature is RF, which is necessary for the diagnosis of rheumatism and it is where the largest gap occurs, followed by C3. C3 is important in diseases such as lupus because complement levels are altered under these conditions; hence, C3 becomes critical. Other second-level technical features, such as ANA, anti-Ro, anti-La, anti-dsDNA, and anti-SM autoantibodies, contribute less strongly but they are important in the diagnosis of certain autoimmune diseases, such as AS and SLE. Hypertension, which is driven by age and sex, is not critical, at least in measuring disease risk or poor health outcomes. Since clinical and laboratory biomarkers, especially those of inflammation and active tubular autoantibodies, are more common than are demographic biomarkers. This gives credence to two of our clinical tests in ESR, RF, C3, anti-CCP, and CRP, as first-line diagnostic tools for autoimmune diseases.

The global feature importance using GBoost model as shows in Fig. 11 reveals that ESR and RF are dominant predictors, aligning with current diagnostic guidelines where these markers are first-line tests for inflammatory arthritis. However, the model additionally highlights C3 as equally important as RF (scores 0.35 *vs*. 0.34), suggesting lab workflows might prioritize C3 testing more frequently than currently done a potentially practice changing insight.

#### Comparative analysis

The datasets in the ML approach often encounter the common challenge of considering class imbalance, where models overlearn a particular class and neglect minority classes, which makes their performance low on such classes. To overcome this, data balancing techniques, which involve oversampling, are applied. It is crucial to assess the effectiveness of such techniques by performing a performance comparison on the initial set, which was parched, and the resulting balanced set. The direct difference between the balanced and unbalanced metrics can be expressed as the absolute difference (AD), as shown in Eq. (18). A positive value means that there is an improvement within the bias correction method, and a negative value means that there is a reduction within the method. The percentage difference (PD) is used to represent the improvement or degradation of the performance after the dataset has been balanced over the values of the unbalanced performance and it is represented mathematically in Eq. (19).

(18) $$AD = Balance\_dataset\mbox{-}Unbalance\_dataset$$

(19) $$PD = {{AD} \over {Unbalanced\_dataset}}*100.$$

Table 20 shows the performance of the different metrics of the ML models with a greater focus on the AD and PD between the balanced and unbalanced datasets. Models like KNN and SVM showed significant improvements, with KNN achieving a 15.14% precision PD, while RF and ET displayed minimal changes. The stochastic behavior of SGD was evident, with metrics fluctuating significantly, indicating inconsistent adaptation to dataset balancing. AdaBoost and NB demonstrated moderate enhancements, with PD values ranging from approximately 4% to 8% when handling highly enhanced dataset imbalances. The present results highlight that appropriately balancing the training datasets helps enhance model performance. However, the extent of the improvements is dependent on the models.

**Table 20:** Performance comparison of ML models for Unbalanced and Balanced dataset, showing AD and PD for seven evaluation metrics.

Model	Precision		Recall		F-score		Kappa		HL		MCC		Accuracy	
	AD	PD	AD	PD	AD	PD	AD	PD	AD	PD	AD	PD	AD	PD
XGBoost	3.304	3.97%	5.192	6.41%	4.458	5.45%	4.936	6.27%	−3.892	−21.91%	4.914	6.24%	3.892	4.73%
KNN	9.468	15.14%	9.107	14.69%	8.877	14.37%	7.099	12.07%	−5.554	−16.20%	7.490	12.70%	5.554	8.45%
DT	4.785	6.07%	4.746	6.02%	4.792	6.08%	4.321	5.72%	−3.375	−16.52%	4.314	5.71%	3.375	4.24%
RF	0.203	0.24%	5.050	6.27%	3.317	4.03%	2.891	3.61%	−2.136	−12.97%	2.886	3.59%	2.136	2.56%
SVM	12.333	19.64%	9.101	14.08%	9.780	15.40%	3.749	5.70%	−2.610	−9.21%	4.027	6.10%	2.610	3.64%
ANN	5.358	6.87%	5.361	6.98%	4.924	6.37%	2.870	3.80%	−2.149	−10.54%	3.155	4.18%	2.149	2.70%
SGD	2.202	3.06%	−1.956	−2.75%	−2.641	−3.85%	−3.728	−5.60%	3.296	11.81%	−3.348	−4.94%	−3.296	−4.57%
GBoost	0.803	0.93%	4.744	5.78%	3.259	3.90%	3.846	4.77%	−2.967	−18.45%	3.844	4.76%	2.967	3.54%
LGBM	2.755	3.30%	4.565	5.61%	3.819	4.65%	4.391	5.55%	−3.291	−19.04%	4.327	5.46%	3.291	3.98%
AdaBoost	3.539	4.45%	5.046	6.48%	4.546	5.80%	4.275	5.68%	−3.613	−17.44%	4.157	5.51%	3.613	4.56%
NB	4.117	6.17%	5.143	7.88%	4.723	7.17%	5.359	8.49%	−4.490	−12.93%	5.405	8.55%	4.490	6.88%
ET	−0.179	−0.21%	2.689	3.22%	1.355	1.59%	2.895	3.49%	−1.964	−11.83%	2.805	3.38%	1.964	2.36%

**Figure 12:**
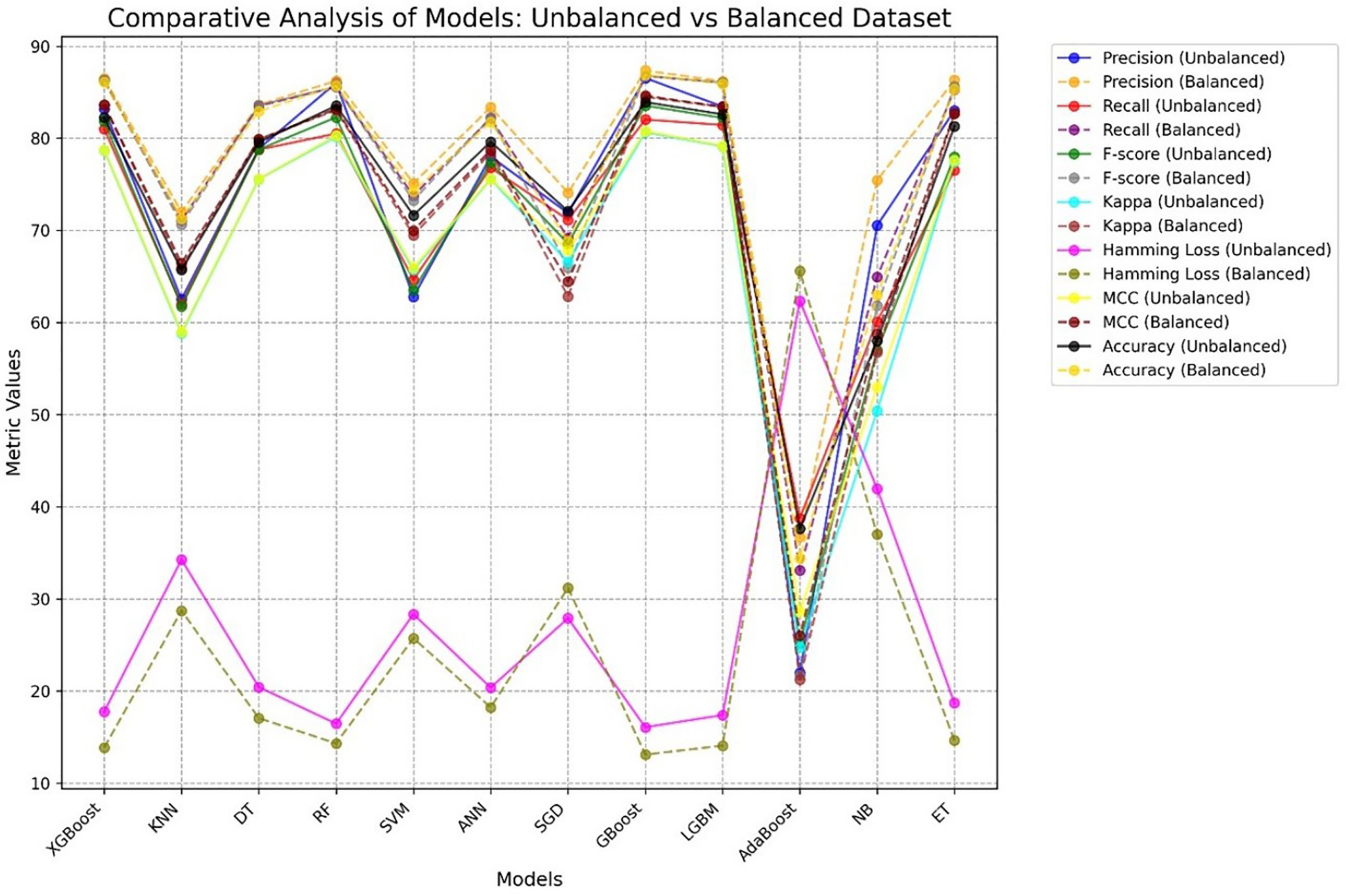
Comparative performance analysis of 12 ML models using balanced *vs*. unbalanced datasets, showing AD and PD across seven evaluation metrics.

Figure 12 presents a comparative analysis of different ML procedures with respect to unbalanced and balanced datasets. The performance of most models generally increases when the datasets are balanced, especially for the minority class, and the precision-recall trade-off increases with increasing MCC and F-score for balanced datasets.

The least effective algorithm in this study is AdaBoost which recorded a very low accuracy ranging from 34% to 37%. This dismal outcome can be linked to a number of reasons: (i) highly correlated of features which was likely to confuse the weak learners and hence perform poorly, (ii) a restricted number of weak learners (set to 50 in this investigation) which may prove to be too few to learn the patterns that exist in the data, and (iii) the inclusion of noise in the form of irrelevant features in the dataset.

Table 21 achieve marginally higher accuracy (*e.g*., Wu et al., 2025; Wang et al., 2023; Jia et al., 2024), our study distinguishes itself in several key areas that make it more robust, generalizable, and clinically relevant. Most prior studies are limited to binary classification (*e.g*., RA *vs*. non-RA), whereas our proposed framework simultaneously classifies six distinct rheumatic and autoimmune diseases with normal case, providing a more realistic and useful tool for differential diagnosis. Our dataset comprises 12,085 real patient records, significantly surpassing synthetic datasets (Martins et al., 2023) and small image dataset (Nouira et al., 2024). This enhances the model’s applicability in actual clinical settings. Our proposed work use FDOSM which allows to choose best ML model from 12. We integrate LIME to provide local interpretability of predictions, a critical factor for adoption in healthcare environments where transparency is essential. With an accuracy of 86.89%, our proposed maintains high predictive performance while offering explainability and multi-class support, balancing performance with interpretability something rarely achieved together in the literature.

**Table 21:** Summary of studies on disease diagnosis with ML methods.

Ref	Disease(s)	Method	Data size	Accuracy (%)
Martins et al. (2023)	ALS, MS, SLE, CD, AIH	LR, NB, SVM	Synthetic (1,000)	84
Wang et al. (2023)	LN	XGB	1,467	99
Park (2024)	SLE	GB, RF	24,990	88
Pham et al. (2024)	RA	XGB	5,600	81.6
Jia et al. (2024)	AS	SCJAYA-FKNN	UCI + AS	99.2
Nouira et al. (2024)	SS	CNN	225	99
Qiu et al. (2025)	RA	LightGBM	SHARE survey	90.2
Wu et al. (2025)	RA	RF, LR	2,106 + external	96.2
Our work	6 diseases with normal	12 ML models + FDOSM + LIME	12,085	86.89

This work addresses the pressing challenge of accurately diagnosing complex and overlapping autoimmune and rheumatic diseases, which are often misclassified due to the lack of specific biomarkers and the subjective interpretation of clinical signs. Traditional ML models may offer high accuracy, but they often lack transparency and reliability in critical domains like healthcare.

To tackle this, we proposed a hybrid framework that combines multiple ML models and FDOSM with XAI. The FDOSM enables a multi-criteria evaluation that goes beyond accuracy to include precision, recall, kappa, and other metrics, incorporating expert judgment in a rational and fair ranking process. Meanwhile, XAI enhances trust and clinical adoption by offering global and local explanations for each diagnosis, thereby supporting transparent and evidence-based medical decisions.

The theoretical contribution of this study lies in demonstrating how FDOSM and XAI can bridge the gap between predictive accuracy and interpretability. Our results show that GBoost algorithm is top performer according to FDOSM. Also, more interpretable when coupled with XAI. This dual strength significantly contributes to the emerging field of trustworthy AI in healthcare.

This work also has limitations such as; (i) The dataset was exclusively collected from Iraq (2019–2024), which may include (patient demographics, genetic, and healthcare protocols), (ii) investigating how the use of deep learning-based imputation may influence the results by remaining more robust, (iii) investigating whether any of the models, such as feature selection or feature reduction, could provide insight into which variables are more valuable in predicting the results of the model, and (iv) the absence of adversarial testing: the models were not evaluated against adversarial attacks. There are a number of possible fixes that might be used to overcome these limitations.

– Future studies should incorporate datasets from multi-center, international cohorts to enhance generalizability.
– Sophistication imputation methods, including denoising autoencoders are used to handle complex missing data patterns. These strategies may produce better imputed values and capture underlying data distributions.
– To identify the most important variables for model prediction, t-SNE, PCA, or recursive feature elimination (RFE) is used.
– Adversarial testing techniques, such as Projected Gradient Descent (PGD) or the Fast Gradient Sign Method (FGSM), are used to assess the model’s resistance to adversarial attacks.

## Policy suggestions

The proposed framework for diagnosing rheumatic and autoimmune diseases demonstrates significant potential to improve clinical decision-making. To translate these findings into real-world impact, we recommend the following policies:

– Healthcare institutions should pilot the top performing algorithm which is GBoost in regions with high disease prevalence (*e.g*., Iraq, where the dataset was collected).
– Fund multicenter collaborations to expand datasets for rare conditions (*e.g*., ReA, which had only 516 cases before balancing). Data collection protocols should mandate the inclusion of all 14 clinical features identified in Table 2 to ensure model compatibility.
– Clinicians should be trained to interpret local and global XAI, Figs. 8 to 11 to build trust in AI-assisted diagnoses. Prioritize biomarkers identified as critical by the GBoost model (*e.g*., ESR, RF, C3, and anti-CCP) as shown in Fig. 11 in routine screenings to enable early detection.
– Policymakers should enforce guidelines for complete EHR documentation to reduce missing values (*e.g*., 43% missing in Anti-Sm) as shown in Table 10. Mandate ADASYN algorithm (preprocessing) in AI training pipelines to ensure equitable performance across minority classes
– Highlight the role of AI in early diagnosis to encourage timely testing for high-risk groups.

## Conclusion

This work proposes a new diagnostic method that employs a combination of ML and FDOSM with XAI to enhance the diagnosis of rheumatic and autoimmune diseases. This framework has an overlapping and vague nature of its symptoms. The FDOSM model indicates that the GBoost algorithm was the best-performing model among 12 ML models, with an accuracy rate of 86.889%. To support clinical applicability, the framework incorporates XAI techniques that provide both local and global interpretability, thereby increasing transparency and fostering trust in real-world diagnostic settings. This framework demonstrates significant advancements; several limitations are acknowledged. The dataset, collected from Iraqi healthcare centers between 2019 and 2024, may reflect regional biases in healthcare practices and patient demographics, potentially limiting generalizability. Additionally, certain biomarkers exhibited high rates of missing values, which, despite the use of imputation methods, could have influenced model performance. To mitigate these issues, future research should involve multi-center collaborations across diverse regions and adopt prospective data collection strategies to enhance robustness and external validity. To advance the framework’s utility, future work may incorporate more sophisticated imputation approaches such as deep learning-based imputation (*e.g*., denoising autoencoders), as well as dimensionality reduction techniques like t-SNE, PCA, or RFE to optimize feature selection. Furthermore, adversarial robustness should be assessed using techniques like PGD or FGSM to ensure model stability under challenging conditions. The potential use of transfer learning and domain adaptation can also address domain-specific biases in the data. Despite these challenges, this research offers a promising step forward. It not only provides a comprehensive and explainable diagnostic tool but also contributes the first known tabular dataset covering seven types of rheumatic and autoimmune diseases, made openly available to support further research. By combining ML interpretability with fuzzy evaluation, the proposed framework sets a strong foundation for improving diagnostic accuracy, enhancing clinical decision support, and ultimately contributing to better patient outcomes.

## Supplemental Information

10.7717/peerj-cs.3096/supp-1 Supplemental Information 1 Rheumatic and Autoimmune Diseases Dataset.

10.7717/peerj-cs.3096/supp-2 Supplemental Information 2 Code for all algorithms, including plots.

10.7717/peerj-cs.3096/supp-3 Supplemental Information 3 Code for Explainable Artificial Intelligence.

10.7717/peerj-cs.3096/supp-4 Supplemental Information 4 Pipline code for all algorithms, including plots.

10.7717/peerj-cs.3096/supp-5 Supplemental Information 5 Principal Component Analysis.

## Additional Information and Declarations

### Data Availability

The following information was supplied regarding data availability:

The data is available at Harvard Dataverse: Dhafar Hamed Abd; Mohammed Fadhil Mahdi; Arezoo Jahani, 2025, “Rheumatic and autoimmune diseases dataset”, https://doi.org/10.7910/DVN/VM4OR3, Harvard Dataverse, V3.
